# Temporal dynamics of spontaneous default-mode network activity mediate the association between reappraisal and depression

**DOI:** 10.1093/scan/nsy092

**Published:** 2018-10-19

**Authors:** Wei Gao, ShengDong Chen, Bharat Biswal, Xu Lei, JiaJin Yuan

**Affiliations:** 1 The Laboratory for Affect Cognition and Regulation (ACRLAB), Key Laboratory of Cognition and Personality of Ministry of Education (SWU), Faculty of Psychology, Southwest University, Chongqing, China; 2 Department of Biomedical Engineering, New Jersey Institute of Technology, Newark, NJ, USA; 3 Sleep and Neuroimaging Center, Faculty of Psychology, Southwest University, Chongqing, China

**Keywords:** emotion regulation, reappraisal, default mode network, Hurst, depression

## Abstract

Cognitive reappraisal is associated with major depressive disorder (MDD), while spontaneous activity patterns of the default mode network (DMN) is implicated in reappraisal and MDD. However, neural mechanisms subserving the close association of spontaneous reappraisal and depression are unclear. Spontaneous reappraisal, depression and resting-state functional magnetic resonance imaging (rsfMRI) were measured from 105 healthy subjects. We assessed the temporal complexity (Hurst exponent), Regional Homogeneity (ReHo) and fractional Amplitude of Low Frequency Fluctuation (fALFF) profiles of DMN, a network involved in both reappraisal and depression. Mediation effects of these standard measures on the relationship between reappraisal and depression, and the contributions of each DMN subregion, were assessed. Results indicated that Hurst exponent (H) of DMN, whether extracted by independent component analysis (ICA) or region of interest (ROI), was significantly associated with reappraisal scores. An individual with a higher reappraisal score has a lower Hurst value of DMN. Mediation analyses suggest that H of DMN partially mediates the association between reappraisal and the degree of depression, and this mediation effect arises from the contribution of medial prefrontal cortex. Neither ReHo nor fALFF showed a similar correlation or mediation effect. These findings suggest that temporal dynamics of DMN play an important role in emotion regulation and its association with depression. H of DMN may serve as a neural marker mediating the association between reappraisal and depression.

Cognitive reappraisal refers to changing the way one thinks about a potentially emotion-eliciting situation to regulate its emotional impact (Buhle *et al*., 2014). Several studies have demonstrated reappraisal is effective at reducing subjective, behavioral and physiological aspects of emotions (Egloff *et al*.*,*^2006^; Johnco *et al*., 2014). Multiple studies have shown that cognitive reappraisal is associated with default mode network (DMN) regions that are implicated in self-referential processing and emotional appraisal (Abler *et al*., 2010; Sambataro *et al*., 2013; Vanderhasselt *et al*., 2013; Lau *et al*., 2015; Martins and Mather, 2016). For instance, key nodes of DMN have been implicated in successful reappraisal (Ochsner *et al*., 2012; Uchida *et al*., 2015). In addition, DMN increases its activity and functional connectivity with key nodes of the emotional circuit (e.g. the right amygdala and insula) during cognitive reappraisal relative to passive viewing of negative stimuli (Sripada *et al*., 2014; Xie *et al*., 2016). These evidences suggest that DMN plays an important role in cognitive reappraisal process.

Recently, there is increasing interest on spontaneous (or habitual) reappraisal (Ehring *et al*., 2010; Volokhov and Demaree, 2010; Quigley and Dobson, 2014; Samson *et al*., 2015). Distinct from instructed, task-driven reappraisal, spontaneous reappraisal arises spontaneously, without explicit instructions from another person, as in most cases of daily-life reappraisal. Several studies have demonstrated spontaneous reappraisal can occur during resting state without any explicit requirement of emotion regulation (Disner *et al*., 2011; Liao *et al*., 2011; Ertl *et al*., 2013; Uchida *et al*., 2015). It has been established that spontaneous reappraisal serves a predictor for depression (Martin and Dahlen, 2005; Abler *et al*., 2010; Min *et al*., 2013). Specifically, Joormann and Gotlib (2010) found that individual differences in the use of reappraisal play an important role in depression, with less reappraisal use, predicting higher levels of depressive symptoms. DMN function is considered to subserve individual differences in reappraisal. For instance, Uchida *et al*. (2015) found that lesser resting-state functional connectivity (RSFC) between right amygdala and two nodes of DMN [i.e. medial prefrontal cortex (MPFC) and posterior cingulate cortex (PCC)] predicted greater reappraisal success. Also, studies indicate that individual differences in spontaneous reappraisal affect DMN’s intrinsic functional connectivity at rest (Martins and Mather, 2016; Morawetz *et al*., 2016). Therefore, it is plausible that DMN’s activity at rest is associated with individual differences in spontaneous reappraisal.

Furthermore, many studies have shown a close relationship between reappraisal, DMN activity and depressive disorder (Johnstone *et al*., 2007; Sheline *et al*., 2009; Shi *et al*., 2015; Wei *et al*., 2015). The neuroimaging research on reappraisal indicates less effective modulation of emotion-processing regions by key nodes of DMN in major depressive disorder (MDD). One study found greater activation of temporal pole and dorsal cingulate in MDD adults compared with controls during reappraisal (Beauregard *et al*., 2006). A separate study of MDD adults reported that during reappraisal of emotional pictures, non-depressed individuals, but not their depressed counterparts, displayed both increased dorsolateral prefrontal cortex (DLPFC) activation and decreased amygdala activation, mediated by activity in the ventromedial prefrontal cortex (Johnstone *et al*., 2007). Together, these findings suggest that MDD is characterized by increased activation of emotional reactivity regions during reappraisal of negative emotions and that this reactivity may be associated with abnormal function of DMN, leading to deficient emotional regulation. Moreover, one study of individual difference found that in contrast to the association of expressive suppression with higher self-reported symptoms, cognitive reappraisal was associated with lower levels of depression in the undergraduate sample (Moore *et al*., 2008). Therefore, it is possible that the relationship between spontaneous reappraisal and depression is associated with DMN’s activity.

As stated, previous studies have studied DMN-reappraisal association in terms of functional connectivity between brain regions. However, reappraisal is a process unfolding over time, entailing breakdown of stereotyped cognitive schema and reformulation of a new evaluation (Koval *et al*., 2015). In this regard, the temporal dynamics of brain activation should be pivotal in the representation of emotion regulation, which was confirmed by substantial studies (Thiruchselvam *et al*., 2011; Paul *et al*., 2013; Pavlov *et al*., 2014; Koval *et al*., 2015). Since the mid-1990s, the dynamics of the brain at rest has been attracting a growing body of research in neuroscience. Neuroimaging studies have revealed distinct functional networks that slowly activate and deactivate, pointing to the existence of an underlying network dynamics emerging spontaneously during rest (Fox *et al*., 2007; Deco *et al*., 2011; Ciuciu *et al*., 2012). Several studies have already shown that DMN dynamics is disrupted in depression (Hamilton *et al.*, 2011; Wei *et al*., 2015; Kaiser *et al.*, 2016), and abnormal dynamic RSFC in MDD was associated with medial prefrontal and temporal regions which involved in emotion regulation processing, such as reappraisal (Dillon and Pizzagalli, 2013; Murphy *et al*., 2016). For instance, Kaiser’s findings indicate that depression was related to decreased dynamic (less variable) RSFC between MPFC and other regions of the prototypical default network, but increased dynamic (more variable) RSFC between MPFC and regions of insula and lateral prefrontal cortex (Murphy *et al*., 2016). In the present study, we examine the relationship between reappraisal, DMN activity and depression by the Hurst exponent, a single numerical quantity indicating the behavior of the autocorrelation function of a monofractal time series (He *et al*., 2010; He, 2011; Ciuciu, *et al*., 2012).

The Hurst exponent was applied to blood oxygen level-dependent (BOLD) signal measured under both physiological and pathological conditions (Wink *et al*., 2006; Lai *et al*., 2010; Lei *et al*., 2013; Gentili *et al*., 2015). The original value of H ranges continuously between 0 and 1. Specifically, H closer to 0.5 indicates more randomness or chaos (e.g. Brownian motion) whereas the H value closer to 1 indicates more regular or persistent fluctuations (e.g. Euclidian order). A value of 0.5 < H < 1 represents positively autocorrelated or persistent behavior; while 0 < H < 0.5 demonstrates negatively autocorrelated or anti-persistent behavior; H = 0.5 corresponds to classical Gaussian white noise. It means that the time course is a random white noise series when Hurst exponent is equal or close to 0.5. Previous studies found that DMN exhibited smaller Hurst exponent in MDD as compared with healthy controls (Wei *et al*., 2013; Wei *et al*., 2015). In these studies, Wei and colleagues extracted the weight vectors defined by the distance to the hyperplane to observe the effect of different features on the classification. Specifically, the Hurst exponent corresponding to resting-state networks was represented by the weight vectors in MDD *vs* healthy control comparison, in order to extract classification features with the support vector machine (SVM) approach. SVM achieved good discriminative performance and effectively identified MDD patients as shown by this study (Wei *et al*., 2013). However, in the present study, we aim to explore individual differences in DMN in order to account for the relationship between spontaneous reappraisal and depression. Therefore, extracting original values of H, which ranges from 0–1 continuously, is more appropriate to our current study instead of using the weight vectors of DMN.

By using original values of H, previous evidences showed that individual with high social anxiety and introversion has a higher Hurst exponent (Lei *et al*., 2013; Gentili *et al*., 2015), and a number of studies have demonstrated that individual with high social anxiety and introversion has a higher depression score (Janssonfröjmark and Lindblom, 2008; Uliaszek *et al*., 2010; Grav *et al*., 2012; Shanahan *et al*., 2014). For instance, Lei and colleagues showed an inverse relationship between H in DMN and extraversion (Lei *et al*., 2013). And Gentili and colleagues found a positive correlation between the H and the social anxiety scores in DMN regions including the posterior cingulate, the precuneus and bilateral inferior parietal sulcus (Gentili *et al*., 2015). Additionally, severity of autistic symptoms was negatively correlated with H in retrosplenial and right anterior insular cortex (Lai *et al*., 2010). However, these brain regions are not involved in DMN. Relatively, Maxim and colleagues demonstrated that patients with early Alzheimer’s disease had greater persistence of rsfMRI noise (larger H) in medial and lateral temporal cortex, dorsal cingulate and premotor cortex, and left pre- and post-central gyrus which are involved in DMN (Maxim *et al*., 2005). Therefore, we hypothesize that a higher original value in Hurst exponent of DMN corresponds to a higher risk of depression.

In contrast to the temporal dynamic profiles depicted by the Hurst exponent, other standard rsfMRI measures like Regional Homogeneity (Reho) and fractional Amplitude of Low Frequency Fluctuation (fALFF) depict complementary information about network integrity and both have been found to be useful in characterizing regional alterations (Denier *et al*., 2015; Xu *et al*., 2015). More specifically, fALFF is thought to reflect the strength of spontaneous neural activation (Chao *et al*., 2017; Zou *et al*., 2008) while ReHo represents the local synchronicity of neural activations in the same functional cluster (Zang *et al*., 2004; Jiang and Zuo, 2016), both irrelevant to temporal dynamic features. And given the close association between spontaneous reappraisal and resting-state DMN function and that between DMN and depression, we hypothesize that the resting-state functioning of DMN may be an important neural mechanism underpinning the association between spontaneous reappraisal and depression. This hypothesis was tested by a line of mediation analyses in the present study, including Hurst exponent, ReHo and fALFF measures of resting-state DMN. According to aforementioned analyses, we predict that temporal dynamics of DMN measured by Hurst exponent, rather than other resting state measures, may mediate the association between spontaneous reappraisal and depression. Finally, we computed the Hurst exponent in the subregions of DMN and evaluated the contributions of each subregion to the mediation, respectively.

## Methods and Materials

### Participants and individual difference measures

A total of 110 right-handed, healthy college students participated in the study (58 females; mean age = 21.13 years, s.d. = 1.58). Five participants were excluded (excessive head movement, >2 mm) and 105 participants were included in the final analysis (55 females; mean age = 21.04, s.d. = 1.52). All participants gave written informed consent and were paid for their participation. This study was approved by the local ethical committee of Southwest University and the Institutional Human Participants Review Board of the Southwest University Imaging Center for human brain research. Individual differences in everyday use of reappraisal measures were administered before fMRI scanning. The depression scale that we used was the Beck’s Depression Inventory (Beck *et al*., 1961). We controlled for individual differences in emotion reactivity by assessing neuroticism and trait anxiety and controlled for the use of another common strategy to downregulate emotion by assessing suppression. The primary measure of interest was the reappraisal scale of the Emotion Regulation Questionnaire (ERQ; Gross and John, 2003). This scale consists of 10 items designed to assess individual differences in reappraisal (six) use (e.g. `I control my emotions by changing the way I think about the situation I’m in’). This scale has been shown to have good internal consistency and test–retest reliability and to be independent from intelligence and socioeconomic statues (Gross and John, 2003). Control measures also included (i) the Chinese version of 48-item Neuroticism questionnaire of the Neuroticism-Extraversion-Openness (NEO) Five-Factor Personality Inventory (Szymkowicz *et al*., 2016) which assesses individual’s preference to experience psychological distress; (ii) the trait version of the State Trait Anxiety Inventory (STAI trait version; Spielberger *et al*., 1970) which assesses individual differences in trait anxiety; and (iii) the suppression scale of the ERQ which assesses individual differences in the use of suppression.

### fMRI data analysis

#### Data acquisition

rsfMRI data were acquired with a Siemens 3 T scanner (Siemens Magnetom Trio TIM, Erlangen, Germany). Each scan contains 232 functional volumes (about 5 min), collected with an Echo-planar imaging (EPI) sequence (TR = 2 s, TE = 30 ms, flip angle = 75°, matrix size = 64 × 64, FoV = 220 × 220 mm2, voxel size = 3.4 × 3.4 × 3 mm3, Slices = 32). Anatomical images were also collected for normalization with a T1-weighted protocol (TR = 1900 ms, TE = 2.52 ms, FA = 9°, matrix = 64 × 64, FoV = 256 × 256 mm2, voxel size = 1 × 1 × 1 mm3). All subjects were instructed to fixate on the center of the screen, not to think about or concentrate on anything in particular and to remain as motionless as possible. Head movements were minimized by using a cushioned head fixation device.

**Fig. 1:**
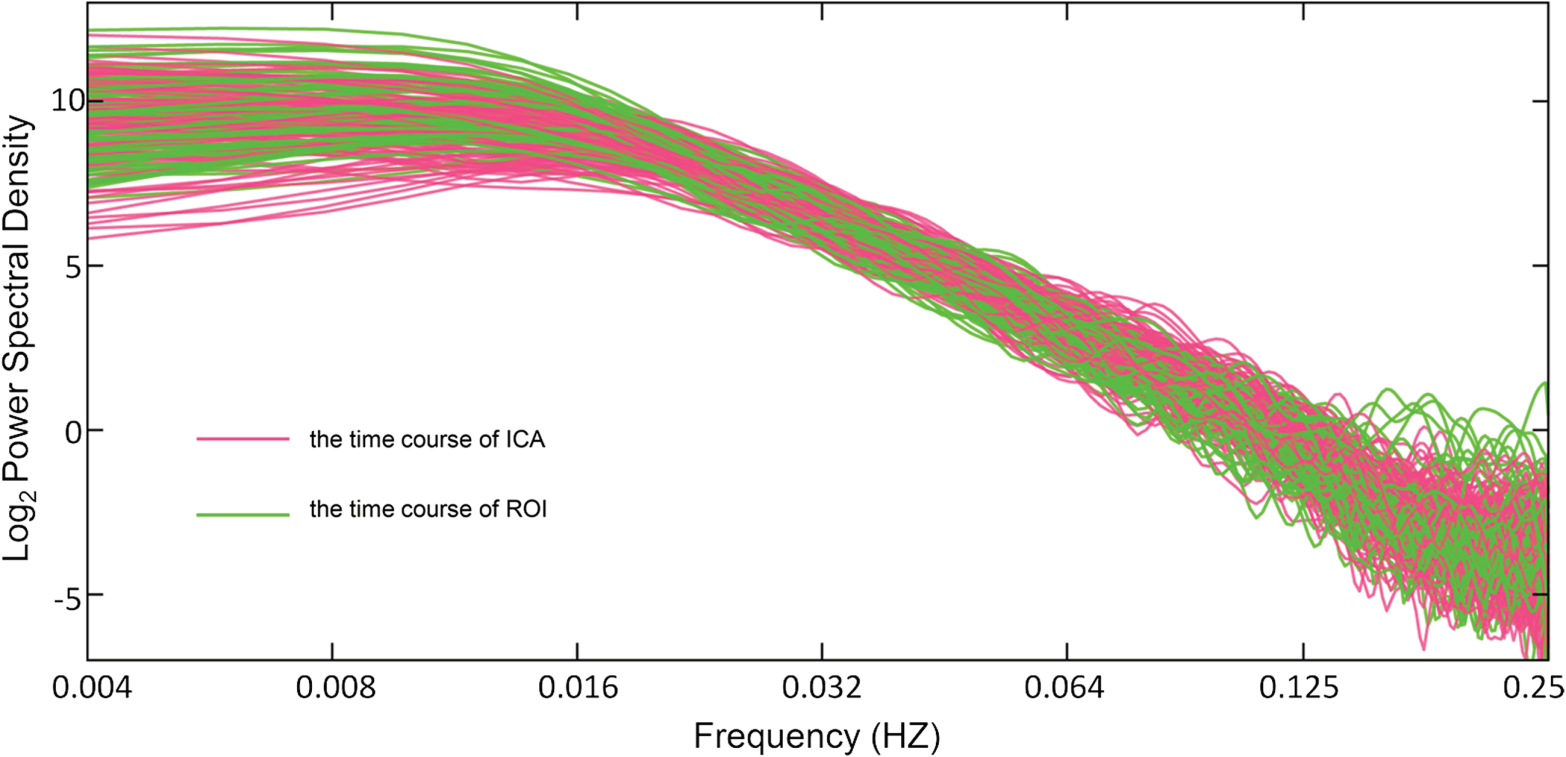
The power spectra of all subjects without band-pass filtering from the cumulative sum of the time courses of ICA (red) and region of interests (ROIs; green). The scale parameters were chosen to correspond to a frequency range of (0.016, 0.063) Hz.

#### Preprocessing

FMRI data were preprocessed and analyzed using Data Processing Assistant for rsfMRI Advanced Edition (DPARSFA v3.2, http://rfmri.org/DPARSF) (Yan *et al*., 2016) of Data Processing & Analysis of Brain Imaging (DPABI v1.3, http://rfmri.org/DPABI) toolbox and SPM8 software (http://www.fil.ion.ucl.ac.uk/spm) implemented in Matlab (R2010a, processing MathWorks, Inc., USA). The first 10 volumes of each functional time series were discarded to allow for scanner stabilization. The remaining functional images were initially corrected for within-scan acquisition time differences between slices. In order to remove motion related confounds (Satterthwaite *et al*., 2013; Power *et al*., 2014; Power *et al*., 2015; Siegel *et al*., 2016), we performed motion correction using SPM’s realign function to align each individual’s BOLD fMRI data to the mean of the images. During motion correction, head movement was recorded in six directions and used to exclude individuals with significant motion (2 mm) and to regress out the effects of motion on BOLD signal. Following motion correction, each individual’s rsfMRI data were coregistered to their corresponding anatomical image. Each anatomical image was segmented into gray matter, white matter (WM) and cerebrospinal fluid (CSF) probability maps using the ‘New Segment’ function in SPM8 while deriving a deformation field. Following segmentation individual’s rsfMRI data were transformed to MNI standard space using the deformation field derived during the segmentation step. For all individuals, probability maps for CSF and WM were thresholded at *P* > 0.95 to create CSF and WM masks, respectively. Using these masks, the BOLD time series were extracted from the resting-state dataset and the first five principal components were derived. A principal component analysis (PCA)-based noise correction (CompCorr) and Friston-24-based generalized linear model (GLM) model (Friston *et al*., 1996) was implemented to reduce the effect of physiological noise and motion time series from the BOLD fMRI data using DPABI. The GLM model thus included a total of 34 regressor time series (5 principal components of WM, 5 principal components of CSF, 6 motion parameters, 6 autoregressive motion parameters and 12 quadratic models of the motion parameters). Finally, normalized functional images were resliced into 3 × 3 × 3 mm3 voxels and spatially smoothed with a Gaussian kernel (8 mm FWHM).

#### DMN identification with group independent component analysis

The preprocessed data from all the subjects were analyzed with group ICA (GIFT, http://icatb.sourceforge.net/). The optimal number of principal components was set as 30, using the GIFT dimensionality estimation tool. First, data from each subject was reduced using PCA, according to the selected number of components. Second, the data was separated by ICA using the Extended Infomax algorithm. Third, independent components (ICs) and time courses for each subject were back-reconstructed and the mean spatial maps for each group were transformed to Z-scores for display. The IC that best matched DMN as previously reported (Greicius and Menon, 2004) was selected and the corresponding time course DMN_ICA_ was used to generate the Hurst exponent estimation (Figure 1).

#### DMN identification with seed at PCC

Seed-based analysis was also used for defining DMN as an attempt to avoid potential selection effects of defining DMN. A single spherical region (radius 10 mm) positioned in the PCC (0, −52, 30) was selected as the seed (Fransson, 2005). Cross-correlation analysis was performed by computing temporal correlation between the mean time course of the seed and BOLD signal intensity of all brain voxels. Correlation coefficients of each voxel were normalized to Z-scores with Fisher’s *r* to *z* transformation and thus an entire brain Z-score map was created for each subject. The mean signal of all the voxels with a Z-score larger than 3 was regarded as the time course DMN_ROI_, and was input for Hurst exponent estimation (Figure 1).

**Fig. 2:**
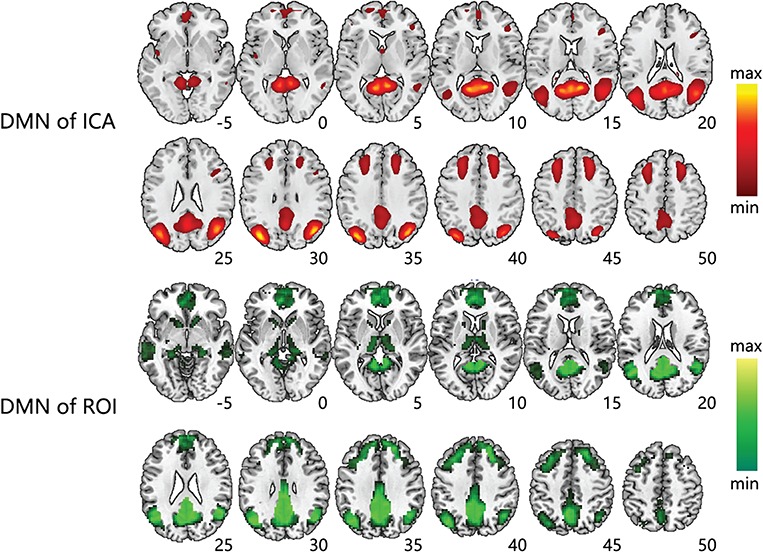
The spatial patterns of DMN extracted by ICA and ROI. DMN extracted by group ICA (Top) and DMN extracted by ROI seeded in PCC region (Bottom). Axial map was shown to be superimposed on the MNI152 standard space template image. The statistical thresholds of both spatial patterns were *P* < 0.05 (FDR-corrected, 20 adjacent voxels).

#### Hurst exponent.

The scale parameters were chosen to correspond to a frequency range of (0.016, 0.063) Hz (Ciuciu *et al*., 2012). For the log–log scale plot of the power spectra, the linear slope (B) is used to calculate the Hurst exponent by the formula H = (B − 1) / 2 (Figure 2). Further analyses were conducted using Matlab 11.0 (Math Works, Natick, MA) and the Hurst exponent was calculated from DMN_ICA_ and DMN_ROI_ using the WLBMF toolbox (https://www.irit.fr/∼Herwig.Wendt/). For details on our parameters, see Supplementary Table S1. Finally, the Spearman rank correlations were calculated between the Hurst exponent and individuals’ scores on ERQ. Including the age and gender as covariates led to comparable results.

#### fAFLL and ReHo

Other standard measures (fALFF, ReHo) used in resting state MRI analysis were also calculated in this study. ReHo reflects the temporal homogeneity of the regional BOLD signal and fALFF is the fraction of ALFF in a given frequency band to the ALFF over the entire frequency range detectable in the given signal (Zou *et al*., 2008). ReHo and fALFF analyses were performed by using the rsfMRI Data Analysis Toolkit (http://resting-fmri.sourceforge.net).

#### Analysis of DMN subregions

The default network comprises a set of interconnected brain regions, including MPFC, PCC and medial temporal lobe (MTL). Though these subregions, from a point of functional integration, constitutes an organized network during rest (Greicius and Menon, 2003; Fox *et al*., 2005), increasing evidence has suggested greater heterogeneity within DMN than is commonly appreciated (Roy *et al*., 2009). Therefore, it is possible that the components of DMN may play different roles in emotion regulation process. To address this issue, we also examined whether the H of DMN at subregion level may mediate individual differences in spontaneous reappraisal and their association with depression. We divided DMN into three key nodes (Andrews-Hanna, 2012) by the ‘Free ROI’ tool (http://freeroi.brainactivityatlas.org).We then extracted the time courses and calculated the Hurst exponent of MPFC, PCC and MTL separately. Finally, H of three key nodes of DMN was involved in the subsequent correlation and mediation analysis.

**Fig. 3:**
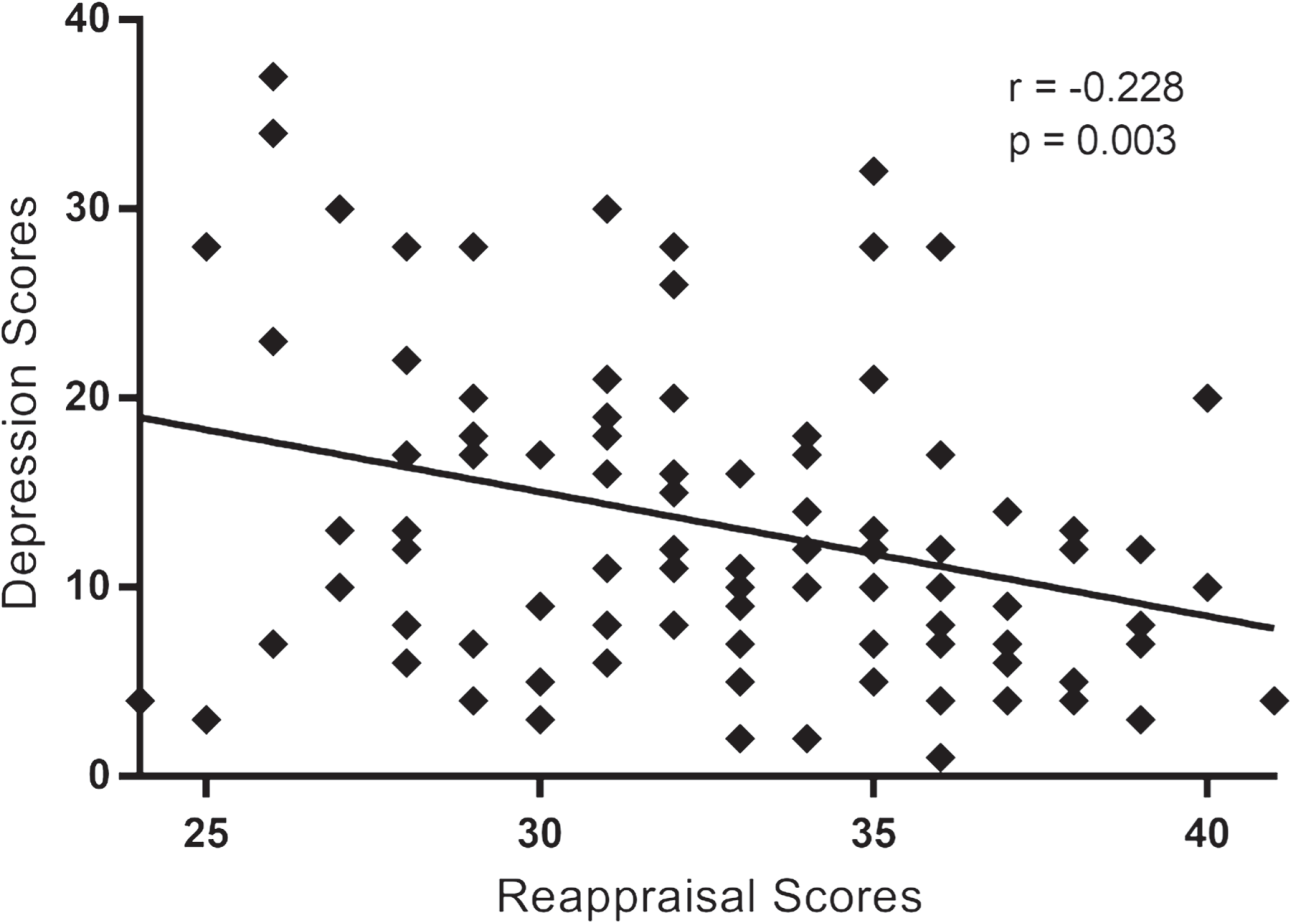
Spearman rank-correlations and scatter-plots displaying the relationship between the scores of reappraisal and depression scores. Significant negative correlation between reappraisal and depression scores, *r* (105) = −0.288; *P* < 0.01.

**Fig. 4:**
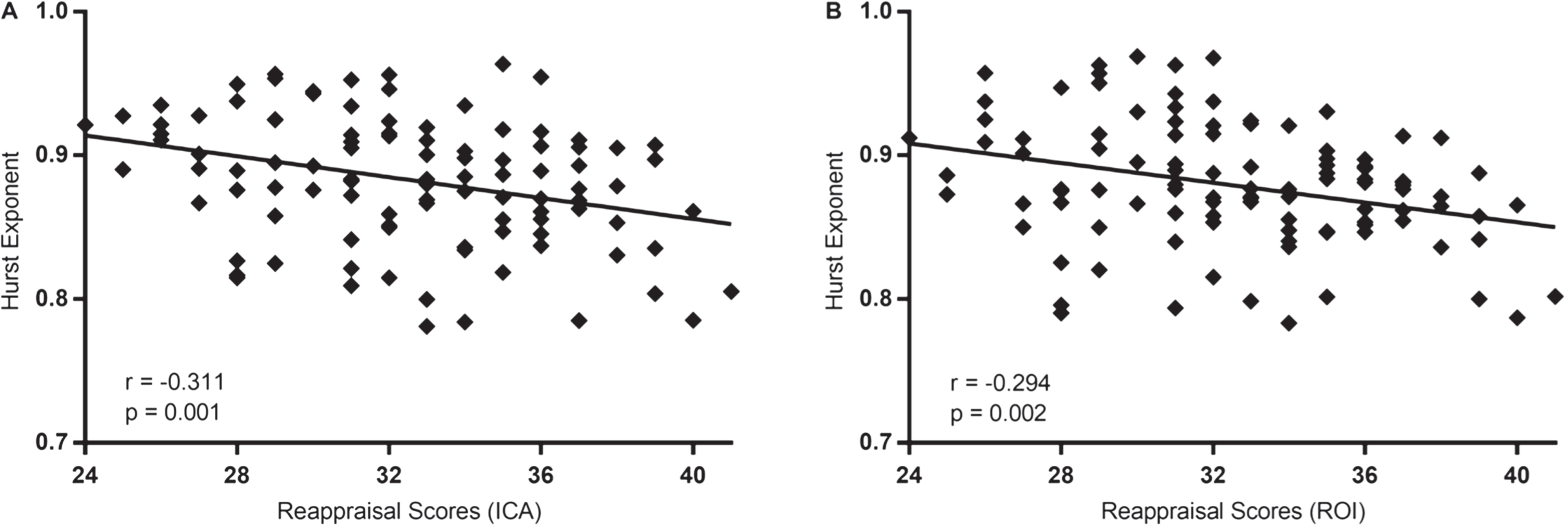
Spearman rank correlations and scatter plots displaying the relationship between the scores of reappraisal and the Hurst exponents in DMN_ICA_**(A)** and in DMN_ROI_**(B)**. Significant negative correlation between reappraisal scores and H of DMN_ICA_, *r* (105) = −0.311; *P* < 0.01; H of DMN_ROI_, *r* (105) = −0.294; *P* < 0.01.

## Results

### Spatial patterns of DMN

The correlation analysis showed significant negative correlations between reappraisal and depression scores, *r* (105) = −0.288; *P* = 0.003 (Figure 3). Consistent with previous studies Martin and Dahlen, 2005; Abler *et al*., 2010; Min *et al*., 2013), our current result showed that an individual with a higher reappraisal score had a lower depression score.

The correlation analysis showed significant negative correlations between reappraisal scores and H of DMN_ICA_, *r* (105) = −0.311; *P* = 0.001 (Figure 4A). Thus, an individual with a higher reappraisal score had a lower Hurst value of DMN_ICA_. However, no significant correlations were found between suppression scores and H of DMN_ICA_, *r* (105) = −0.01, *P* = 0.918. These results indicated that during rest, the temporal complexity of DMN was associated with the use of reappraisal, but not with the use of suppression. Of note, the alternative approach, calculating the correlation between the ERQ scores and H of DMN_ROI_, yielded a similar result that the H of DMN_ROI_ was significantly associated with the use of reappraisal, *r* (105) = −0.294, *P* = 0.002 (Figure 4B), but not with the use of suppression *r* (105) = −0.03, *P* = 0.725.

Moreover, given the heterogeneity within DMN, we also examined the relationship between Hurst in key components of DMN and the use of reappraisal. Results showed that the use of reappraisal, but not the use of suppression, was negatively associated with H of PCC, H of MPFC and H of MTL, respectively (Table 1. For more details on fALFF/ReHo, see Supplementary Table S1). These findings suggest that the Hurst of both DMN and its components may serve as a specific neuroimaging marker of the spontaneous and uninstructed reappraisal.

### Specificity of findings

Previous studies demonstrated that extraversion and trait anxiety are correlated with Hurst exponent in DMN (Lei *et al*., 2013; Gentili *et al*., 2015) and reappraisal (Martin and Dahlen, 2005; Uliaszek *et al*., 2010). In order to ensure our findings independent of emotional reactivity (Gentili *et al*., 2015) and personality trait (Lei *et al*., 2013), we used a hierarchical regression analysis to test (i) whether extraversion and trait anxiety scores were related to H of DMN and its components and (ii) whether the relationship between reappraisal and H of DMN (including its components) withstood correction for these factors. Regression analyses on H of DMN showed that 14.4% of the variance in the criterion variable was accounted for by the statistical model (F (3, 101) = 5.661; *P* < 0.001; Table 2). Reappraisal captured 9.7% of the variance while the addition of trait anxiety and extraversion to the equation did not result in a significant increment of R^2^. These findings indicated that our primary results were not due to individual differences in emotional reactivity or personality trait, and specific to reappraisal.

**Table 1:** The correlation between resting-state measurements and reappraisal/suppression scores

	Reappraisal	Suppression
H of PCC	*r* = −0.208; *P* = 0.033	*r* = −0.012; *P* = 0.900
H of MPFC	*r* = −0.241; *P* = 0.013	*r* = 0.111; *P* = 0.260
H of MTL	*r* = −0.210; *P* = 0.031	*r* = 0.065; *P* = 0.510
fALFF of DMN	*r* = 0.020; *P* = 0.840	*r* = 0.133; *P* = 0.175
ReHo of DMN	*r* = −0.065; *P* = 0.509	*r* = 0.087; *P* = 0.376

**Table 2:** Regression coefficients (R^2^, △R^2^) and statistical results of hierarchical linear regression analyses on H of DMN with respect to the influence of reappraisal, trait anxiety and extraversion are shown

**Dependent Variables**	**Step**	**H of DMN**			
		Beta	R^2^	△R^2^	*P*<
reappraisal			0.097		0.001
	reappraisal alone	−0.312		0.097	0.001
reappraisal, TA			0.114		0.002
	reappraisal added first	−0.275		0.097	0.005
	TA added second	0.135		0.017	0.166
reappraisal, TA, extraversion			0.144		0.001
	reappraisal added first	−0.263		0.097	0.007
	TA added second	0.136		0.017	0.159
	extraversion added third	−0.172		0.030	0.065

**Note:** TA, trait anxiety. Probability values are two tailed. R^2^ illustrates the regression model, whereas △R^2^ illustrates the improvement of the regression model when additional independent variables are considered.

Other standard measures (fALFF, ReHo) used in resting state MRI measures were also calculated using these indexes. No significant correlations were found between fALFF/ReHo of DMN and reappraisal scores (Table 1). Therefore, the association between DMN and spontaneous reappraisal is specific to the Hurst index of this network, as other standard measures (fALFF, ReHo) cannot reflect the relationship between DMN activity and spontaneous reappraisal. Previous studies have indicated that fALFF and ReHo help to reveal the complexity of the brain function, by reflecting the intensity and homogeneity of regional spontaneous brain activity, respectively (Zang *et al*., 2004; Zou *et al*., 2008). However, these indicators cannot reflect the temporal dynamic property of spontaneous brain activity. Therefore, our current results suggested that temporal scale of spontaneous brain signals may play a more important role in emotion regulation process and depression.

### H of DMN mediates the association of reappraisal with depression

**Fig. 5:**
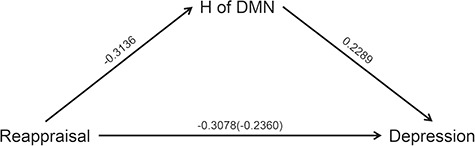
The relationship between reappraisal and depression scores was mediated by H of DMN. The lines are labeled with path coefficients and standard errors are shown in parentheses. The direct path between spontaneous reappraisal and depression is calculated controlling for H. Indirect path *a* = −0.31136, indirect path *b* = 0.2289, total relationship *c* = −0.3078 and direct path *c* = −0.2360. The values in parentheses indicate the strength of the path prior to the inclusion of the mediating variable, ^*^P < 0 .05.

The mediation analysis is based on a standard three-variable path model and with a bootstrap test for the statistical significance of the indirect effect, as diagrammed in Figure 5. As illustrated in Figure 5, the results showed that the indirect effects of the use of reappraisal on the subjective depression scores were significant (H of DMN_ICA_: a^*^b = −0.0718,*P* < 0.05; H of DMN_ROI_: a^*^b = −0.1023, *P* < 0.05). But further mediation analysis results of three subregions of DMN showed that only H of MPFC mediates the association between the use of reappraisal and depression. For details on our mediation analysis, see Supplementary Table S1. These results, shown in Table 3, indicate that resting-state temporal complexity in DMN explains a part of the reappraisal and depression association.

## Discussion

Most literature on DMN-reappraisal association to date focused on the functional connectivity between brain regions. The present fMRI study explored the temporal dynamics of DMN during resting state and examined the association between the Hurst exponent of DMN and the ERQ scores. Our findings indicate that more use of spontaneous reappraisal predicts less memory of resting-state neural signals (i.e. Hurst exponents closer to 0.5) in DMN, regardless of whether the Hurst exponent was extracted by ROI or ICA. Importantly, further mediation analysis indicated that the temporal dynamics of DMN (particularly the MPFC) measured by Hurst exponent mediate the association between spontaneous reappraisal and depression. Our findings suggest that temporal scale of spontaneous brain signals may play an important role in emotion regulation process and its association with depression.

In the present study, we found that a higher Hurst exponent of DMN corresponds to a higher risk of depression. This appears inconsistent with previous findings that DMN exhibited decreased Hurst value in MDD as compared with healthy controls (Wei *et al*., 2013; Wei *et al*., 2015). We consider that two reasons may contribute to this difference. On the one hand, as stated before, different algorithms were used to represent Hurst exponent in the above and the current studies, driven by different research purposes. We used original Hurst value that ranges continuously from 0–1, while Wei and colleagues used weight vectors in support vector machine approach, to represent the H. On the other hand, individual differences in healthy population, as involved in the current study, may be reflected by distinct DMN temporal dynamic profiles from those in clinical population. Specifically, Wei and colleagues considered that the decreased H may suggest irregular regional oscillation originated from persistent negative thoughts in MDD. However, previous studies demonstrated that there are different behaviors and brain mechanisms between MDD and healthy people (Wang *et al*., 2015; Kaiser *et al*., 2016; Tozzi *et al*., 2017). For instance, Kaiser *et al*., 2016 found that MDD with ruminative thinking have abnormal patterns of fluctuating communication among brain systems compared with healthy people. In this regard, healthy population, who have no symptoms of persistent negative thoughts and ruminative coping (Zetsche *et al.*, 2012; D’Avanzato *et al.*, 2013), may have distinct individual difference profiles in the temporal dynamics of DMN; that is, the implication of individual differences in Hurst exponent of DMN should be considered in the context of different populations. The relationship between emotion regulation strategy and Hurst exponent of DMN in depressed patients should be examined in future studies.

The Hurst exponent is a useful measure to characterize different physiological states, as shown multiple times (Berthouze *et al*., 2010; Bojic and Vuckovic, 2010; Ciuciu *et al*., 2012; Tagliazucchi *et al*., 2013; Kantelhardt *et al*., 2015; Churchill *et al*., 2016). According to previous evidences, a smaller value of H suggests the brain network is more efficient in online information processing and less in long-range memory (Yang and Tsai, 2013). However, a recent study suggests that the stationary fractional Gaussian noise (fGn) process is not sufficient to describe neural data (Von *et al*., 2018). So the interpretation of memory effects in real-world signals may benefit from information-theoretical analyses, in addition to Hurst exponent estimation. As a result, the interpretation that the long-range memory can be predicted by Hurst exponents correctly should be more cautious.

Resting-state studies of spontaneous fluctuations in fMRI signals have demonstrated huge potential in mapping the brain’s intrinsic functional features (Krüger and Glover, 2001; Yan *et al*., 2010). Ciuciu *et al*., 2012 found that spontaneous brain activity exhibits scale-free dynamics, suggesting the temporal complexity and fractal-like of the resting-state BOLD signal. In previous study, researchers quantified the temporal complexity of rsfMRI based on H, because H can reflect the property of scale-free dynamics via describing the self-similarity of time courses (Maxim *et al.*, 2005; Park *et al.*, 2010). The complexity of resting-state BOLD signals could provide some evidence of dynamics of intrinsic brain activity (Yang *et al.*, 2013). Wink *et al.* utilized H to quantify fractal complexity and describe pathological and physiological features, then found that normal aging is accompanied by a loss of complexity (decreased H) in bilateral hippocampus. Therefore, in the present study, the negative correlation between reappraisal and H of DMN may suggest that participant with a higher reappraisal score (a lower H value) is associated with increase of complexity in the DMN. Recently, Dong *et al.*, 2018 used the H exponent to explore fractal complexity of the rsfMRI signal in the human brain across the adult lifespan. And they found a significant positive correlation between the mean H of whole-brain gray matter and the age of all subjects, suggesting that H increases with age. That is, complexity of BOLD activity is reduced with age. Further, their results showed that healthy aging is accompanied by reduced complexity (increased H) in frontal and parietal lobes and by increased complexity (decreased H) in insula, limbic and temporal lobes. They speculate that age-related increase of complexity in insula is because insula is critical for emotional feeling (Gasquoine, 2014), and with aging, the adult’s ability to regulate emotion remains stable and improves in some aspects (Nashiro *et al*., 2012). Previous studies have suggested that the DMN is critical for self-referential processing, affective cognition and emotion regulation (Buckner *et al*., 2008; Andrewshanna *et al*., 2010). Therefore, it is reasonable that increase of complexity (decreased H) in the DMN is associated with a higher reappraisal score.

**Table 3:** Mediation effects of H of DMN in the prediction of depression (N = 105)

	Depression	
Mediator	Point estimate (a^*^b)	Bootstrapping BC 95% CI
H of DMN_ICA_	−0.0718	[−0.1651, −0.0112]
H of DMN_ROI_	−0.0691	[−0.1706, −0.0105]
H of PCC	−0.0180	[−0.0818, 0.0306]
H of MPFC	−0.0788	[−0.1897, −0.0175]
H of MTL	0.0218	[−0.0147, 0.0871]

**Note:** The mediation effects of the use of reappraisal on subjective depression were bootstrapped using 5000 samples. Approximately 95% bias-corrected confidence intervals for all indirect effects and contrasts were generated.

Prior studies have indicated a close association between information processing efficiency in the brain and one’s cognitive flexibility (Blackwell *et al*., 2009; Wingenfeld *et al*., 2011; Ragozzino *et al.*, 2012). In this regard, cognitive flexibility might be another interpretation for our results. Cognitive flexibility is defined as the ability to adjust one’s emotional, cognitive and behavioral responses to a situation based on new information (Martin and Rubin, 1995; Johnco *et al.*, 2014). Cognitive flexibility predicts effective reappraisal of emotion situation (Malooly, 2012) and is closely linked to rumination [i.e. failure to shift thoughts away from a past threat (Nolen-Hoeksema, 2000; Joormann *et al.*, 2011; Nolen-Hoeksema, 2000)] and worry [i.e. failure to shift thoughts away from future threat (Lee and Orsillo, 2013)]. It has been reported that cognitive inflexibility is associated with difficulty in adjusting cognition, attitude and behavior despite the input of new information (Hamtiaux and Houssemand, 2012). Previous studies have demonstrated that cognitive flexibility is associated with depression and depressed patients have impaired cognitive flexibility (Joormann and Siemer, 2011; Murphy *et al*., 2012); that is, similar to the Hurst of DMN that mediates the association between reappraisal and depression, cognitive flexibility also predicts both reappraisal and depression. On the other hand, the Hurst exponent reflects the temporal complexity of neural activity patterns. Low complexity is analogous to the continuation of the established cognitive schema over time, as reflected by cognitive inflexibility. Based on these considerations, we posit that a higher Hurst may correspond to greater cognitive inflexibility; that is, people with a higher Hurst value (worse online information processing), are less effective in reappraisal and at higher risk of depression, most likely as a result of cognitive inflexibility. However, caution should be exercised during inferring the Hurst of DMN to cognitive flexibility, as this variable was not directly assessed in the current study. This potential association needs to be addressed in future studies.

The mediation analysis showed that the relationship between spontaneous reappraisal use and depression is mediated by H from DMN, particularly the MPFC. Several previous studies have shown that MPFC is generally involved in cognitive control and has important relation with the successful reappraisal during emotion regulation (Ridderinkhof *et al*., 2004; Etkin *et al*., 2006; Goldin *et al*., 2008; Buhle *et al.*, 2014). Goldin *et al.* (2008) suggest that MPFC regions play a crucial role in downregulating neural responses of emotion-generating regions to negative stimuli, like amygdala and insula. Many studies have suggested that the other two subregions in DMN are involved in both memory and emotion. There is considerable evidence that the MTL has functions related to episodic or remote autobiographical memories (Simons and Spiers, 2003; Phelps, 2004; Buchanan *et al*., 2005; Aggleton, 2012) and the PCC shows increased activity when individuals retrieve autobiographical memories or plan for the future (Mantani *et al.*, 2005; Addis *et al*., 2007; Mason *et al.*, 2007; Leech and Sharp, 2013). Together with the function of three subregions in DMN mentioned in the previous paragraph, our results indicate that people who use reappraisal more (lower Hurst value) tend to have a greater capability of online information processing, which facilitate the cognitive control of MPFC. What’s more, a shorter long-range memory in PCC or MTL can help people to ruminate less but more engage in online cognitive activity, which facilitate successful reappraisal.

However, further mediation analysis showed that only the temporal complexity of MPFC mediates the relationship between reappraisal use and depression. One possible explanation is that MPFC is involved in both cognitive control and self-referential processing. MPFC is critical in internal, self-referential processing (Northoff *et al.*, 2006) and has been suggested to play an important role in self-referential processing in major depression (Mitchell *et al*., 2005; Lemogne *et al.*, 2010; Lemogne *et al*., 2012). Lemogne *et al.* (2012) has provided a compelling evidence for the role of an increased MPFC activity in the depressive self-focus which is associated with acute depressive states and with an increased risk of depressive relapse through ruminative processes. Additionally, previous studies have shown that successful performance of cognitive flexibility depends on the normal structure and function of MPFC (Dalley *et al*., 2004; Hang *et al.*, 2016). For instance, both lesions in MPFC and alterations of the level of MPFC dopamine have been found to induce disturbances in cognitive flexibility (Logue and Gould, 2014; Hernandez *et al.*, 2016). The Hurst exponent has been proposed as a measure of online information-processing efficiency: lower Hurst values are related to lower temporal redundancy and more freedom to vary (cognitive flexibility; He, 2011). Therefore, we suggest that differences of the temporal complexity in spontaneous MPFC activity may represent a possible neurobiological correlate of cognitive flexibility. Together, we suggest a loss of complexity in MPFC may reflect a decreased ability of cognitive control, more rumination on self-referential memories and cognitive inflexibility, which causes vulnerability to depression.

### Clinical implications

There is an increasing consensus that dysfunctional emotion regulation is a core feature of major psychiatric illnesses like depression or anxiety disorders (Martin and Dahlen, 2005; Abler *et al.*, 2010; Min *et al.*, 2013). Decreased use of reappraisal in particular, as indexed by the ERQ, is associated with increased depressive symptoms (Gross and John, 2003). In addition, a series of studies have shown that dysfunction of the default-mode network is associated with depression (Johnstone *et al.*, 2007; Sheline *et al.*, 2009; Shi *et al.*, 2015; Wei *et al.*, 2015). Our results suggest the temporal complexity in DMN plays a crucial mediating role in the relationship between the use of reappraisal and depression. This suggests that people who use reappraisal less (higher Hurst value) tend to have a loss of complexity of neural signals in DMN, implying reduced cognitive flexibility (and difficulty in adapting to environmental changes). Those people are more likely to ruminate in the negative context, which causes them to fall into stronger depression. The present study, therefore, offers a consideration on depression treatment by changing the temporal complexity in DMN, for example, by the reappraisal training.

### Limitations and future directions

The study has five limitations that necessitate future investigation and research. First, only rest-state data were studied. Therefore, it is possible that the results of this study cannot be generalized to task activity studies. Future studies should combine with emotion regulation task to further study this association. Second, although the association between H of DMN and the use of reappraisal has been examined by different methods and appear to be robust, these findings are limited only to the index of H of DMN. Therefore, future studies should apply multiple approaches to delineate brain network dynamics like sliding window methods (Kaiser *et al.*, 2016), co-activation pattern analysis (Chen *et al*., 2015) and multi-layered dynamic analysis (Raz *et al.*, 2012) to further confirm the observed associations in follow-up studies. Third, only healthy young participants were studied, and it is therefore unknown whether our results are generalizable to all age groups. Because age differences are often observed in studies of emotion regulation (Martins *et al*., 2016; Scheibe *et al*., 2015), it is important to examine whether there is an association between emotion regulation tendencies and age differences. Fourth, MDD patients are not involved in the current study. Thus, individual differences in temporal dynamics of DMN in MDD patients and its association with emotion regulation tendencies should be examined in future studies. Finally, recent study has found that the mutual information function of neurophysiological data behaves differently from fGn, and the H phenomenon is a sufficient condition to prove long-range memory only in the stationary fGn process (Von *et al*., 2018). Thus, the interpretation that the long-range memory can be predicted by Hurst exponents correctly should be more cautious, and future study should use the time-lagged mutual information function (a novel and effective tool to assess long-range dependence in finite length empirical data) as a complementary method to measure memory effects.

## Conclusion

In conclusion, the present study provides first evidence for a negative association between the use of cognitive reappraisal and the temporal dependence of DMN during resting state. Specifically, individual with a higher reappraisal score has a lower Hurst value of DMN as shown by the resting-state functional MRI. More importantly, our mediation results suggest that the temporal complexity of DMN plays an important role in the relationship between spontaneous reappraisal and depression, which shed light on depression intervention in the field of clinical practice. These findings suggest that H of DMN at rest may serve as a neuroimaging marker of one’s spontaneous, uninstructed reappraisal and its association with depression.

## Funding

This scientific work was supported by the National Natural Science Foundation of China (Nos 30870668, 81273674 to J.Y.).

*Conflict of interest*. All authors report no biomedical financial interests or potential conflicts of interest.

## Supplementary Material

Supplementary Data
