# Supplementary material for: Temporal dynamics of spontaneous default-mode network activity mediate the association between reappraisal and depression
Source: Soc Cogn Affect Neurosci. 2018 Oct 19;13(12):1235–47. doi: 10.1093/scan/nsy092 (PMC6277739; doi:10.1093/scan/nsy092)
Supplement: Supplementary Data [file nsy092_supp.zip › scan-18-121-File001.docx]

**Reviewer: 1**

**Comments to the Author**

**Although it’s clear that the authors put a lot of work into this revision and attempting to respond to our comments, I am still generally skeptical of the findings.**

**1. It is still not clear how the authors’ contributions fit into the already murky mixed evidence concerning the hurst exponent and personality/psychopathology. The authors did not explain why the papers I cited previously found what they did and instead relied on two papers on extraversion/social anxiety to form the foundation of their entire paper. It’s hard not to get the impression that researchers throw a lot of people in the scanner, give them a bunch of personality scales and see what sticks. I’m not saying that is what the researchers did, but the wild discrepancies between the patterns of the relationships between H and personality/psychopathology among the different studies needs to be fully explicated. Otherwise, it’s hard not to come to that conclusion.**

**Response:**

We greatly appreciate your comments. Following your suggestions, we carefully reviewed previous studies related to the relationships between H and personality/psychopathology among the different studies again. We found that two factors may lead to mixed evidences concerning the H exponent and personality/psychopathology: different research method and different brain location.

First, in research methods, Wei and colleagues extracted the weight vectors defined by the distance to the hyperplane to observe the effect of different features on the classification. Wei’s studies take the values of H corresponding to resting-state networks as classification features, support vector machine (SVM) achieved good discriminative performance and correctly identified MDD patients. On the other hand, some recent studies have used original values of H to explore individual differences in personality/psychopathology, researchers extracted original values of H and calculated the correlation coefficient between H and personality trait scores. And they found that individual with high social anxiety and introversion has a higher Hurst exponent (Gentili et al., 2015; Lei et al., 2013). In the present study, we aim to explore individual differences in DMN in order to account for the relationship between spontaneous reappraisal and depression. Therefore, extracting original values of H, which ranges from 0 to 1 continuously, is more appropriate to our current study instead of using the weight vectors of DMN.

Second, Lai and colleagues found that severity of autistic symptoms was negatively correlated with H in retrosplenial and right anterior insular cortex (Lai et al., 2010). And a reduction of H at the left Inferior Frontal Gyrus has been observed in schizophrenic patients (Sokunbi et al., 2014). However, these brain regions are not involved in DMN. Maxim and colleagues demonstrated that patients with early Alzheimer’s disease (AD) had greater persistence of resting fMRI noise (larger H) in DMN regions including medial and lateral temporal cortex, dorsal cingulate and premotor cortex, and left pre- and postcentral gyrus (Maxim et al., 2005). Although these findings do not warrant the conclusion that a higher H is related to worse brain functioning, at least in DMN, we speculate that a higher H value correspond to a higher risk of depression.

We think different conclusions between Wei’s studies and our current study may be caused by different method or some other reasons (eg. only healthy young participants but not patients were studied in our study). We supplement these explanations in the discussion section. Of course using different method to study MDD patients are better to consolidate our current conclusion. Therefore, we supplement these limitations in our manuscript.

**Revised manuscript: page 4, line 17:**

The Hurst exponent was applied to BOLD (blood oxygen level dependent) signal measured under both physiological and pathological conditions (Gentili et al., 2015; Lai et al., 2010; Lei, Zhao, & Chen, 2013; Wink, Bernard, Salvador, Bullmore, & Suckling, 2006). The original value of H ranges continuously between 0 to 1. Specifically, H closer to 0.5 indicates more randomness or chaos (e.g. Brownian motion) whereas the H value closer to 1 indicates more regular or persistent fluctuations (e.g. Euclidian order). A value of 0.5<H<1 represents positively autocorrelated or persistent behavior; while 0<H<0.5 demonstrates negatively autocorrelated or anti-persistent behavior; H=0.5 corresponds to classical Gaussian white noise. It means that the time course is a random white noise series when Hurst exponent is equal or close to 0.5. Previous studies found that DMN exhibited smaller Hurst exponent in major depressive disorder(MDD) as compared with healthy controls (Wei et al., 2015; Wei et al., 2013). In these studies, Wei and colleagues extracted the weight vectors defined by the distance to the hyperplane to observe the effect of different features on the classification. Specifically, the Hurst exponent corresponding to resting-state networks was represented by the weight vectors in MDD versus healthy control comparison, in order to extract classification features with the support vector machine approach. Support vector machine (SVM) achieved good discriminative performance and effectively identified MDD patients as shown by this study (Wei et al., 2013). However, in the present study, we aim to explore individual differences in DMN in order to account for the relationship between spontaneous reappraisal and depression. Therefore, extracting original values of H, which ranges from 0 to 1 continuously, is more appropriate to our current study instead of using the weight vectors of DMN.

By using original values of H, previous evidences showed that individual with high social anxiety and introversion has a higher Hurst exponent (Gentili et al., 2015; Lei et al., 2013), and a number of studies have demonstrated that individual with high social anxiety and introversion has a higher depression score (Grav, Stordal, Romild, & Hellzen, 2012; Janssonfröjmark & Lindblom, 2008; Shanahan, Copeland, Angold, Bondy, & Costello, 2014; Uliaszek et al., 2010). For instance, Lei and colleagues showed an inverse relationship between H in DMN and extraversion (Lei et al., 2013). And Gentili and colleagues found a positive correlation between the H and social anxiety scores in DMN regions including the posterior cingulate, the precuneus and bilateral inferior parietal sulcus (Gentili et al., 2015). Additionally, severity of autistic symptoms was negatively correlated with H in retrosplenial and right anterior insular cortex (Lai et al., 2010). However, these brain regions are not involved in DMN. Relatively, Maxim and colleagues demonstrated that patients with early Alzheimer’s disease (AD) had greater persistence of resting fMRI noise (larger H) in medial and lateral temporal cortex, dorsal cingulate and premotor cortex, and left pre- and postcentral gyrus which are involved in DMN (Maxim et al., 2005). Therefore, we hypothesize that a higher original value in Hurst exponent of DMN correspond to a higher risk of depression.

**Revised manuscript: page 12, line 14:**

In the present study, we found that a higher Hurst exponent of DMN corresponds to a higher risk of depression. This appears inconsistent with previous findings that DMN exhibited decreased Hurst value in major depressive disorder as compared with healthy controls (Wei et al., 2015; Wei et al., 2013). We consider that two reasons may contribute to this difference. On the one hand, as stated before, different algorithms were used to represent Hurst exponent in the above and the current studies, driven by different research purposes. We used original Hurst value that ranges continuously from 0 to 1, while Wei and colleagues used weight vectors in support vector machine approach, to represent the H. On the other hand, individual differences in healthy population, as involved in the current study, may be reflected by distinct DMN temporal dynamic profiles from those in clinical population. Specifically, Wei and colleagues considered that the decreased H may suggest irregular regional oscillation originated from persistent negative thoughts in MDD. However, previous studies demonstrated that there are different behavior and brain mechanism between MDD and healthy people (Kaiser et al., 2016; Tozzi et al., 2017; Wang et al., 2015). For instance, Kaiser et al. found that MDD with ruminative thinking have abnormal patterns of fluctuating communication among brain systems compared with healthy people (Kaiser et al., 2016). In this regard, healthy population, who have no symptoms of persistent negative thoughts and ruminative coping (D’Avanzato, Joormann, Siemer, & Gotlib, 2013; Zetsche, D'Avanzato, & Joormann, 2012), may have distinct individual difference profiles in the temporal dynamics of DMN. That is, the implication of individual differences in Hurst exponent of DMN should be considered in the context of different populations. The relationship between emotion regulation strategy and Hurst exponent of DMN in depressed patients should be examined in future studies.

**Revised manuscript: page 15, line 12:**

MDD patients is not involved in the current study. Thus, individual differences in temporal dynamics of DMN in MDD patients and its association with emotion regulation tendencies should be examined in future studies.

**2. In response to my comment about the von Wegner article showing that the Hurst exponents may not capture long range memory in timeseries, the authors’ only response is to admit that they may not and promise to do more appropriate analyses later. Doesn’t this invalidate the analyses performed for the current paper? Or at least drastically alter their interpretation?**

**Response:**

Thanks for your valuable advice. According to your suggestion, we reviewed recent papers about the H exponent (Dong et al., 2018). We found H is believed to have the capacity to measure brain activity complexity. A higher H corresponds to a lower fractal dimension, and higher H means lower complexity accordingly. The complexity of resting-state BOLD signals could provide some evidence of dynamics of intrinsic brain activity (Yang et al., 2013). Therefore, in present study the negative correlation between reappraisal and H of DMN may suggest that participant with a higher reappraisal score (a lower H value) is associated with increase of complexity in the DMN. We revise our explanation of H throughout our manuscript. Although we supplement other explanations of H, additional approaches should complement existing methodologies in the future and we add this limitation in our manuscript.

**Revised manuscript: page 12, line 32:**

The Hurst exponent is a useful measure to characterize different physiological states, as shown multiple times (Berthouze, James, & Farmer, 2010; Bojic & Vuckovic, 2010; Churchill et al., 2016; Ciuciu, Varoquaux, Abry, Sadaghiani, & Kleinschmidt, 2012; Kantelhardt, Tismer, Gans, Schumann, & Penzel, 2015; Tagliazucchi et al., 2013). According to previous evidences, a smaller value of H suggests the brain network is more efficient in online information processing and less in long-range memory (Yang & Tsai, 2013). However, a recent study suggests that the stationary fractional Gaussian noise (fGn) process is not sufficient to describe neural data (Von, Laufs, & Tagliazucchi, 2018). So the interpretation of memory effects in real-world signals may benefit from information-theoretical analyses, in addition to Hurst exponent estimation. As a result, the interpretation that the long-range memory can be predicted by Hurst exponents correctly should be more cautious.

Resting state studies of spontaneous fluctuations in fMRI signals have demonstrated huge potential in mapping the brain’s intrinsic functional features (Krüger & Glover, 2001; Yan et al., 2010). Ciuciu et al. found that spontaneous brain activity exhibits scale-free dynamics, suggesting the temporal complexity and fractal-like of the resting-state BOLD signal (Ciuciu et al., 2012). In previous study, researchers quantified the temporal complexity of rs-fMRI based on H, because H can reflect the property of scale-free dynamics via describing the self-similarity of time courses (Maxim et al., 2005; Park, Lazar, Ahn, & Sornborger, 2010). The complexity of resting-state BOLD signals could provide some evidence of dynamics of intrinsic brain activity (Yang et al., 2013). Wink et al. utilized H to quantify fractal complexity and describe pathological and physiological features, then found that normal aging is accompanied by a loss of complexity (decreased H) in bilateral hippocampus. Therefore, in the present study, the negative correlation between reappraisal and H of DMN may suggest that participant with a higher reappraisal score (a lower H value) is associated with increase of complexity in the DMN. Recently, Dong and colleagues used the H exponent to explore fractal complexity of the rs-fMRI signal in the human brain across the adult lifespan (Dong et al., 2018). And they found a significant positive correlation between the mean H of whole brain gray matter and the age of all subjects, suggesting that H increases with age. That is, complexity of BOLD activity is reduced with age. Further, their results showed that healthy aging is accompanied by reduced complexity (increased H) in frontal and parietal lobes and by increased complexity (decreased H) in insula, limbic, and temporal lobes. They speculate that age-related increase of complexity in insula is due to that insula is critical for emotional feeling (Gasquoine, 2014), and with aging, the adult’s ability to regulate emotion remains stable and improves in some aspects (Nashiro, Sakaki, & Mather, 2012). Previous studies have suggested that the DMN is critical for self-referential processing, affective cognition, and emotion regulation (Andrewshanna, Reidler, Sepulcre, Poulin, & Buckner, 2010; Buckner, Andrews-Hanna, & Schacter, 2008). Therefore, it is reasonable that increase of complexity (decreased H) in the DMN is associated with a higher reappraisal score.

**Revised manuscript: page 15, line 14:**

Recent study found that the mutual information function of neurophysiological data behaves differently from fractional Gaussian noise (fGn), and the H phenomenon is a sufficient condition to prove long-range memory only in the stationary fGn process (Von, Laufs, & Tagliazucchi, 2018). Thus, the interpretation that the long-range memory can be predicted by Hurst exponents correctly should be more cautious, and future study should use the time-lagged mutual information function (a novel and effective tool to assess long-range dependence in finite length empirical data) as a complementary method to measure memory effects.

**3. Minor point, but in the regression table, the authors need to show the betas for the reappraisal scale for each model (alone, adding TA, adding extraversion, etc..) not just the betas for the new scales in the model.**

**Table 2** **Regression coefficients (R^2^, △R^2^) and statistical results of hierarchical linear regression analyses on H of DMN with respect to the influence of reappraisal, trait anxiety and extraversion are shown.**

| Dependent Variables Step | | H of DMN | | | |
| --- | --- | --- | --- | --- | --- |
|  |  | Beta R^2^ △R^2^ | | | P< |
| reappraisal | 0.097 0.001 | | | | |
|  | reappraisal alone | -0.312 |  | 0.097 | 0.001 |
| reappraisal, TA | 0.114 0.002 | | | | |
|  | reappraisal added first | -0.275 |  | 0.097 | 0.005 |
|  | TA added second | 0.135 |  | 0.017 | 0.166 |
| reappraisal, TA, extraversion | 0.144 0.001 | | | | |
|  | reappraisal added first | -0.263 |  | 0.097 | 0.007 |
|  | TA added second | 0.136 |  | 0.017 | 0.159 |
|  | extraversion added third | -0.172 |  | 0.030 | 0.065 |

TA: trait anxiety. Probability values are two-tailed. R^2^ illustrates the regression model, whereas △R^2^ illustrates the improvement of the regression model when additional independent variables are considered.

Andrewshanna, J. R., Reidler, J. S., Sepulcre, J., Poulin, R., & Buckner, R. L. (2010). Functional-Anatomic Fractionation of the Brain's Default Network. *Neuron, 65*(4), 550-562.

Berthouze, L., James, L. M., & Farmer, S. F. (2010). Human EEG shows long-range temporal correlations of oscillation amplitude in Theta, Alpha and Beta bands across a wide age range. *Clinical Neurophysiology Official Journal of the International Federation of Clinical Neurophysiology, 121*(8), 1187.

Bojic, T., & Vuckovic, A. A. (2010). Modeling EEG fractal dimension changes in wake and drowsy states in humans--a preliminary study. *Journal of Theoretical Biology, 262*(2), 214-222.

Buckner, R. L., Andrews-Hanna, J. R., & Schacter, D. L. (2008). The brain's default network: anatomy, function, and relevance to disease. *Ann N Y Acad Sci, 1124*, 1-38. doi:10.1196/annals.1440.011

Churchill, N. W., Spring, R., Grady, C., Cimprich, B., Askren, M. K., Reuterlorenz, P. A., . . . Berman, M. G. (2016). The suppression of scale-free fMRI brain dynamics across three different sources of effort: aging, task novelty and task difficulty. *Sci Rep, 6*, 30895.

Ciuciu, P., Varoquaux, G., Abry, P., Sadaghiani, S., & Kleinschmidt, A. (2012). Scale-Free and Multifractal Time Dynamics of fMRI Signals during Rest and Task. *Front Physiol, 3*, 186. doi:10.3389/fphys.2012.00186

D’Avanzato, C., Joormann, J., Siemer, M., & Gotlib, I. H. (2013). Emotion Regulation in Depression and Anxiety: Examining Diagnostic Specificity and Stability of Strategy Use. *Cognitive Therapy and Research, 37*(5), 968-980. doi:10.1007/s10608-013-9537-0

Dong, J., Jing, B., Ma, X., Liu, H., Xiao, M., & Li, H. (2018). Hurst Exponent Analysis of Resting-State fMRI Signal Complexity across the Adult Lifespan. *Frontiers in Neuroscience, 12*, 34.

Expert, P., Lambiotte, R., Chialvo, D. R., Christensen, K., Jensen, H. J., Sharp, D. J., & Turkheimer, F. (2011). Self-similar correlation function in brain resting-state functional magnetic resonance imaging. *Journal of the Royal Society Interface, 8*(57), 472.

Gasquoine, P. G. (2014). Contributions of the Insula to Cognition and Emotion. *Neuropsychology Review, 24*(2), 77.

Gentili, C., Vanello, N., Cristea, I., David, D., Ricciardi, E., & Pietrini, P. (2015). Proneness to social anxiety modulates neural complexity in the absence of exposure: A resting state fMRI study using Hurst exponent. *Psychiatry Res, 232*(2), 135-144. doi:10.1016/j.pscychresns.2015.03.005

Grav, S., Stordal, E., Romild, U. K., & Hellzen, O. (2012). The relationship among neuroticism, extraversion, and depression in the HUNT Study: in relation to age and gender. *Issues Ment Health Nurs, 33*(11), 777-785.

He, B. J. (2014). Scale-free brain activity: past, present, and future. *Trends in Cognitive Sciences, 18*(9), 480-487.

Janssonfröjmark, M., & Lindblom, K. (2008). A bidirectional relationship between anxiety and depression, and insomnia? A prospective study in the general population. *Journal of Psychosomatic Research, 64*(4), 443-449.

Kaiser, R. H., Whitfieldgabrieli, S., Dillon, D. G., Goer, F., Beltzer, M., Minkel, J., . . . Pizzagalli, D. A. (2016). Dynamic Resting-State Functional Connectivity in Major Depression. Neuropsychopharmacology, 41(7), 1822-1830.

Kantelhardt, J. W., Tismer, S., Gans, F., Schumann, A. Y., & Penzel, T. (2015). Scaling behavior of EEG amplitude and frequency time series across sleep stages. *Epl, 112*(1), 18001.

Krüger, G., & Glover, G. H. (2001). Physiological noise in oxygenation-sensitive magnetic resonance imaging. *Magnetic Resonance in Medicine, 46*(4), 631.

Lai, M. C., Lombardo, M. V., Chakrabarti, B., Sadek, S. A., Pasco, G., Wheelwright, S. J., . . . Suckling, J. (2010). A shift to randomness of brain oscillations in people with autism. *Biological Psychiatry, 68*(12), 1092-1099.

Lei, X., Zhao, Z., & Chen, H. (2013). Extraversion is encoded by scale-free dynamics of default mode network. *Neuroimage, 74*, 52-57. doi:10.1016/j.neuroimage.2013.02.020

Maxim, V., Sendur, L., Fadili, J., Suckling, J., Gould, R., Howard, R., & Bullmore, E. (2005). Fractional Gaussian noise, functional MRI and Alzheimer's disease. *Neuroimage, 25*(1), 141-158. doi:10.1016/j.neuroimage.2004.10.044

Nashiro, K., Sakaki, M., & Mather, M. (2012). Age Differences in Brain Activity during Emotion Processing: Reflections of Age-Related Decline or Increased Emotion Regulation? *Gerontology, 58*(2), 156-163.

Park, C., Lazar, N. A., Ahn, J., & Sornborger, A. (2010). A multiscale analysis of the temporal characteristics of resting-state fMRI data. *Journal of Neuroscience Methods, 193*(2), 334-342.

Shanahan, L., Copeland, W. E., Angold, A., Bondy, C. L., & Costello, E. J. (2014). Sleep problems predict and are predicted by generalized anxiety/depression and oppositional defiant disorder. *Journal of the American Academy of Child & Adolescent Psychiatry, 53*(5), 550-558.

Tagliazucchi, E., von Wegner, F., Morzelewski, A., Brodbeck, V., Jahnke, K., & Laufs, H. (2013). Breakdown of long-range temporal dependence in default mode and attention networks during deep sleep. *Proc Natl Acad Sci U S A, 110*(38), 15419-15424. doi:10.1073/pnas.1312848110

Tozzi, L., Doolin, K., Farrel, C., Joseph, S., O'Keane, V., & Frodl, T. (2017). Functional magnetic resonance imaging correlates of emotion recognition and voluntary attentional regulation in depression: A generalized psycho-physiological interaction study. J Affect Disord, 208, 535-544. doi:10.1016/j.jad.2016.10.029

Uliaszek, A. A., Zinbarg, R. E., Mineka, S., Craske, M. G., Sutton, J. M., Griffith, J. W., . . . Hammen, C. (2010). The Role of Neuroticism and Extraversion in the Stress-Anxiety and Stress-Depression Relationships. *Anxiety Stress & Coping, 23*(4), 363.

Von, W. F., Laufs, H., & Tagliazucchi, E. (2018). Mutual information identifies spurious Hurst phenomena in resting state EEG and fMRI data. *Physical Review E, 97*(2-1), 022415.

Wang, K., Wei, D., Yang, J., Xie, P., Hao, X., & Qiu, J. (2015). Individual differences in rumination in healthy and depressive samples: association with brain structure, functional connectivity and depression. Psychol Med, 45(14), 2999-3008. doi:10.1017/S0033291715000938

Wei, M., Qin, J., Yan, R., Bi, K., Liu, C., Yao, Z., & Lu, Q. (2015). Association of resting-state network dysfunction with their dynamics of inter-network interactions in depression. *J Affect Disord, 174*, 527-534. doi:10.1016/j.jad.2014.12.020

Wei, M., Qin, J., Yan, R., Li, H., Yao, Z., & Lu, Q. (2013). Identifying major depressive disorder using Hurst exponent of resting-state brain networks. *Psychiatry Res, 214*(3), 306-312. doi:10.1016/j.pscychresns.2013.09.008

Wink, A. M., Bernard, F., Salvador, R., Bullmore, E., & Suckling, J. (2006). Age and cholinergic effects on hemodynamics and functional coherence of human hippocampus. *Neurobiol Aging, 27*(10), 1395-1404. doi:10.1016/j.neurobiolaging.2005.08.011

Yan, L., Zhuo, Y., Ye, Y., Xie, S. X., An, J., Aguirre, G. K., & Wang, J. (2010). Physiological origin of low-frequency drift in blood oxygen level dependent (BOLD) functional magnetic resonance imaging (fMRI). *Magnetic Resonance in Medicine, 61*(4), 819-827.

Yang, A. C., Huang, C. C., Yeh, H. L., Liu, M. E., Hong, C. J., Tu, P. C., . . . Lin, C. P. (2013). Complexity of spontaneous BOLD activity in default mode network is correlated with cognitive function in normal male elderly: a multiscale entropy analysis. *Neurobiology of Aging, 34*(2), 428-438.

Yang, A. C., & Tsai, S. J. (2013). Is mental illness complex? From behavior to brain. *Prog Neuropsychopharmacol Biol Psychiatry, 45*, 253-257. doi:10.1016/j.pnpbp.2012.09.015

Zetsche, U., D'Avanzato, C., & Joormann, J. (2012). Depression and rumination: relation to components of inhibition. *Cogn Emot, 26*(4), 758-767. doi:10.1080/02699931.2011.613919
