# Supplementary material for: Temporal dynamics of spontaneous default-mode network activity mediate the association between reappraisal and depression
Source: Soc Cogn Affect Neurosci. 2018 Oct 19;13(12):1235–47. doi: 10.1093/scan/nsy092 (PMC6277739; doi:10.1093/scan/nsy092)
Supplement: Supplementary Data [file nsy092_supp.zip › scan-18-121-File002.docx]

Temporal dynamics of spontaneous default-mode network activity mediate the association between reappraisal and depression

Wei Gao^a,*^, ShengDong Chen^a,*^, Bharat Biswal^b^, Xu Lei^c^, JiaJin Yuan^a^

^a^ The Laboratory for Affect Cognition and Regulation (ACRLAB), Key Laboratory of Cognition and Personality of Ministry of Education (SWU), Faculty of Psychology, Southwest University, Chongqing, China

^b^ ^Department of Biomedical Engineering, New Jersey Institute of Technology, Newark, New Jersey^

^c Sleep and Neuroimaging Center, Faculty of Psychology, Southwest University, Chongqing, 400715, China^

^Authors Wei Gao and ShengDong Chen contributed equally to this work.^

^Correspondence to: JiaJin Yuan E-mail:^ [^yuanjiajin168@126.com^](mailto:yuanjiajin168@126.com)

the number of words in the abstract：227

the number of words in the text：6325

the number of tables：3

the number of figures：5

the number of supplementary material：1

**ABSTRACT**

Cognitive reappraisal has been associated with major depressive disorder (MDD) and spontaneous activity patterns of the default mode network (DMN) relate closely with reappraisal and MDD. However, neural mechanisms subserving the close association of spontaneous reappraisal and depression are unclear. Spontaneous reappraisal, depression and resting-state functional magnetic resonance imaging were measured from one-hundred and five healthy subjects. We assessed the temporal complexity (Hurst exponent), Regional Homogeneity (ReHo) and fractional Amplitude of Low Frequency Fluctuation (fALFF) profiles of Default Mode Network (DMN), a network involved in both reappraisal and depression. Mediation effects of these standard measures on the relationship between reappraisal and depression, and the contributions of each DMN subregion, were assessed. Results indicated that H of DMN, whether extracted by independent component analysis (ICA) or region of interest (ROI), was significantly associated with reappraisal scores. An individual with a higher reappraisal score has a lower Hurst value of DMN. Mediation analyses suggest that H of DMN partially mediates the association between reappraisal and the degree of depression, and this mediation effect arises from the contribution of medial prefrontal cortex (MPFC). Neither ReHo nor fALFF showed a similar correlation or mediation effect. These findings suggest that temporal dynamics of DMN play a more important role in emotion regulation and its association with depression. H of DMN may serve a neural marker mediating the association between reappraisal and depression.

***Key words:*** emotion regulation, reappraisal, default mode network, Hurst, depression

Cognitive reappraisal refers to changing the way one thinks about a potentially emotion eliciting situation to regulate its emotional impact (Buhle et al., 2014). Several studies have demonstrated reappraisal is effective at reducing subjective, behavioral, and physiological aspects of emotions (Egloff, Schmukle, Burns, & Schwerdtfeger, 2006; Johnco, Wuthrich, & Rapee, 2014). Multiple studies have shown that cognitive reappraisal is associated with DMN regions that are implicated in self-referential processing and emotional appraisal (Abler et al., 2010; Lau, Leung, Chan, Wong, & Lee, 2015; Martins & Mather, 2016; Sambataro, Wolf, Giusti, Vasic, & Wolf, 2013; Vanderhasselt, Baeken, Van Schuerbeek, Luypaert, & De Raedt, 2013). For instance, key nodes of DMN have been implicated in successful reappraisal (Ochsner, Silvers, & Buhle, 2012; Uchida et al., 2015). In addition, DMN increases its activity and functional connectivity with key nodes of the emotional circuit (e.g. the right amygdala and insula) during cognitive reappraisal relative to passive viewing of negative stimuli (Sripada et al., 2014; Xie et al., 2016). These evidences suggest that DMN plays an important role in cognitive reappraisal process.

Recently, there is increasing interest on spontaneous (or habitual) reappraisal (Ehring, Tuschen-Caffier, Schnulle, Fischer, & Gross, 2010; Quigley & Dobson, 2014; Samson, Hardan, Podell, Phillips, & Gross, 2015; Volokhov & Demaree, 2010). Distinct from instructed, task-driven reappraisal, spontaneous reappraisal arises spontaneously, without explicit instructions from another person, as in most cases of daily-life reappraisal. Several studies have demonstrated spontaneous reappraisal can occur during resting-state without any explicit requirement of emotion regulation (Disner, Beevers, Haigh, & Beck, 2011; Ertl, Hildebrandt, Ourina, Leicht, & Mulert, 2013; Liao et al., 2011; Uchida et al., 2015). It has been established that spontaneous reappraisal serves a predictor for depression (Abler et al., 2010; R. C. Martin & Dahlen, 2005; Min, Yu, Lee, & Chae, 2013). Specifically, Jutta Joormann and Gotlib (2010) found that individual differences in the use of reappraisal play an important role in depression, with less reappraisal use, predicting higher levels of depressive symptoms. DMN function is considered to subserve individual differences in reappraisal. For instance, Uchida et al. (2015) found that lesser resting-state functional connectivity between right amygdala and two nodes of DMN (i.e. MPFC and posterior cingulate cortex) predicted greater reappraisal success. Also, studies indicate that individual differences in spontaneous reappraisal affect DMN’s intrinsic functional connectivity at rest (Martins & Mather, 2016; Morawetz et al., 2016). Therefore, it is plausible that DMN’s activity at rest is associated with individual differences in spontaneous reappraisal.

Furthermore, many studies have shown a close relationship between reappraisal, DMN activity and depressive disorder (Johnstone, van Reekum, Urry, Kalin, & Davidson, 2007; Sheline et al., 2009; Shi et al., 2015; Wei et al., 2015). The neuroimaging research on reappraisal indicates less effective modulation of emotion processing regions by key nodes of DMN in major depressive disorder (MDD). One study found greater activation of temporal pole, and dorsal cingulate in MDD adults compared to controls during reappraisal (Beauregard, Paquette, & Lévesque, 2006). A separate study of MDD adults reported that during reappraisal of emotional pictures, nondepressed individuals, but not their depressed counterparts, displayed both increased DLPFC activation and decreased amygdala activation, mediated by activity in the ventromedial prefrontal cortex (VMPFC) (Johnstone et al., 2007). Together, these findings suggest that MDD is characterized by increased activation of emotional reactivity regions during reappraisal of negative emotions, and that this reactivity may be associated with abnormal function of DMN, leading to deficient emotional regulation. Moreover, one study of individual difference found that in contrast to the association of expressive suppression with higher self-reported symptoms, cognitive reappraisal was associated with lower levels of depression in the undergraduate sample (Moore, Zoellner, & Mollenholt, 2008). Therefore, it is possible that the relationship between spontaneous reappraisal and depression is associated with DMN’s activity.

As stated, previous studies have studied DMN-reappraisal association in terms of functional connectivity between brain regions. However, reappraisal is a process unfolding over time, entailing breakdown of stereotyped cognitive schema and reformulation of a new evaluation (Koval, Butler, Hollenstein, Lanteigne, & Kuppens, 2015). In this regard, the temporal dynamics of brain activation should be pivotal in the representation of emotion regulation, which was confirmed by substantial studies (Koval et al., 2015; Paul, Simon, Kniesche, Kathmann, & Endrass, 2013; Pavlov et al., 2014; Thiruchselvam, Blechert, Sheppes, Rydstrom, & Gross, 2011). Since the mid 1990s, the dynamics of the brain at rest has been attracting a growing body of research in neuroscience. Neuroimaging studies have revealed distinct functional networks that slowly activate and deactivate, pointing to the existence of an underlying network dynamics emerging spontaneously during rest (Ciuciu, Varoquaux, Abry, Sadaghiani, & Kleinschmidt, 2012; Deco, Jirsa, & Mcintosh, 2011; Fox, Snyder, Vincent, & Raichle, 2007). Several studies have already shown that DMN dynamics is disrupted in depression (Hamilton et al., 2011; Kaiser et al., 2016; Wei et al., 2015). And abnormal dynamic resting-state functional connectivity (RSFC) in MDD was associated with medial prefrontal and temporal regions which involved in emotion regulation processing, such as reappraisal (Dillon & Pizzagalli, 2013; E. R. Murphy, Barch, Pagliaccio, Luby, & Belden, 2016). For instance, Kaiser’s findings indicate that depression was related to decreased dynamic (less variable) RSFC between MPFC and other regions of the prototypical default network, but increased dynamic (more variable) RSFC between MPFC and regions of insula and lateral prefrontal cortex (E. R. Murphy et al., 2016). In present study, we examine the relationship between reappraisal, DMN activity and depression by the Hurst exponent, a single numerical quantity indicating the behavior of the autocorrelation function of a monofractal time series (Ciuciu, Varoquaux, Abry, Sadaghiani, Kleinschmidt, et al., 2012; B. J. He, 2011; Biyu J. He, Zempel, Snyder, & Raichle, 2010).

The Hurst exponent was applied to BOLD (blood oxygen level dependent) signal measured under both physiological and pathological conditions (Gentili et al., 2015; Lai et al., 2010; Lei, Zhao, & Chen, 2013; Wink, Bernard, Salvador, Bullmore, & Suckling, 2006). The original value of H ranges continuously between 0 to 1. Specifically, H closer to 0.5 indicates more randomness or chaos (e.g. Brownian motion) whereas the H value closer to 1 indicates more regular or persistent fluctuations (e.g. Euclidian order). A value of 0.5<H<1 represents positively autocorrelated or persistent behavior; while 0<H<0.5 demonstrates negatively autocorrelated or anti-persistent behavior; H=0.5 corresponds to classical Gaussian white noise. It means that the time course is a random white noise series when Hurst exponent is equal or close to 0.5. Previous studies found that DMN exhibited smaller Hurst exponent in major depressive disorder(MDD) as compared with healthy controls (Wei et al., 2015; Wei et al., 2013). In these studies, Wei and colleagues extracted the weight vectors defined by the distance to the hyperplane to observe the effect of different features on the classification. Specifically, the Hurst exponent corresponding to resting-state networks was represented by the weight vectors in MDD versus healthy control comparison, in order to extract classification features with the support vector machine approach. Support vector machine (SVM) achieved good discriminative performance and effectively identified MDD patients as shown by this study (Wei et al., 2013). However, in the present study, we aim to explore individual differences in DMN in order to account for the relationship between spontaneous reappraisal and depression. Therefore, extracting original values of H, which ranges from 0 to 1 continuously, is more appropriate to our current study instead of using the weight vectors of DMN.

By using original values of H, previous evidences showed that individual with high social anxiety and introversion has a higher Hurst exponent (Gentili et al., 2015; Lei et al., 2013), and a number of studies have demonstrated that individual with high social anxiety and introversion has a higher depression score (Grav, Stordal, Romild, & Hellzen, 2012; Janssonfröjmark & Lindblom, 2008; Shanahan, Copeland, Angold, Bondy, & Costello, 2014; Uliaszek et al., 2010). For instance, Lei and colleagues showed an inverse relationship between H in DMN and extraversion (Lei et al., 2013). And Gentili and colleagues found a positive correlation between the H and social anxiety scores in DMN regions including the posterior cingulate, the precuneus and bilateral inferior parietal sulcus (Gentili et al., 2015). Additionally, severity of autistic symptoms was negatively correlated with H in retrosplenial and right anterior insular cortex (Lai et al., 2010). However, these brain regions are not involved in DMN. Relatively, Maxim and colleagues demonstrated that patients with early Alzheimer’s disease (AD) had greater persistence of resting fMRI noise (larger H) in medial and lateral temporal cortex, dorsal cingulate and premotor cortex, and left pre- and postcentral gyrus which are involved in DMN (Maxim et al., 2005). Therefore, we hypothesize that a higher original value in Hurst exponent of DMN correspond to a higher risk of depression.

In contrast to the temporal dynamic profiles depicted by the Hurst exponent, other standard resting-state fMRI measures like Reho and fALFF depict complementary information about network integrity and both have been found to be useful in characterizing regional alterations (Denier et al., 2015; Xu, Zhuo, Qin, Zhu, & Yu, 2015). More specifically, fALFF is thought to reflect the strength of spontaneous neural activation (Chao et al., 2017; Zou et al., 2008) while ReHo represents the local synchronicity of neural activations in the same functional cluster (Jiang & Zuo, 2016; Zang, Jiang, Lu, He, & Tian, 2004), both irrelevant to temporal dynamic features. And given the close association between spontaneous reappraisal and resting-state DMN function and that between DMN and depression, we hypothesize that the resting-state functioning of DMN may be an important neural mechanism underpinning the association between spontaneous reappraisal and depression. This hypothesis was tested by a line of mediation analyses in the present study, including Hurst exponent, ReHo and fALFF measures of resting-state DMN. According to aforementioned analyses, we predict that temporal dynamics of DMN measured by Hurst exponent, rather than other resting state measures, may mediate the association between spontaneous reappraisal and depression. Finally, we computed the Hurst exponent in the subregions of DMN and evaluated the contributions of each subregion to the mediation, respectively.

**METHODS AND MATERIALS**

**Participants and Individual Difference Measures**

A total of 110 right-handed, healthy college students participated in the study (58 females; mean age=21.13 years, SD=1.58). Five participants were excluded (excessive head movement, >2mm) and 105 participants were included in the final analysis (55 females; mean age=21.04, SD=1.52). All participants gave written informed consent and were paid for their participation. This study was approved by the local ethical committee of Southwest University and the Institutional Human Participants Review Board of the Southwest University Imaging Center for human brain research. Individual differences in everyday use of reappraisal measures were administered before fMRI scanning. The depression scale that we used were the Beck's Depression Inventory (BDI) (Beck, Ward, Mendelson, Mock, & Erbaugh, 1961). We controlled for individual differences in emotion reactivity by assessing neuroticism and trait anxiety and controlled for the use of another common strategy to down regulate emotion by assessing suppression. The primary measure of interest was the reappraisal scale of the ERQ (Gross & John, 2003). This scale consists of ten items designed to assess individual differences in reappraisal (six) use (e.g., “I control my emotions by changing the way I think about the situation I’m in”). This scale has been shown to have good internal consistency and test-retest reliability and to be independent from intelligence and socioeconomic statues (Gross & John, 2003). Control measures also included: 1) the Chinese-version of 48-item Neuroticism questionnaire of the NEO Five Factor Personality Inventory (Szymkowicz et al., 2016) which assesses individual’s preference to experience psychological distress; 2) the trait version of the State Trait Anxiety Inventory (STAI trait version) (Spielberger, 1970) which assesses individual differences in trait anxiety. 3) The suppression scale of the ERQ which assesses individual differences in the use of suppression.

**fMRI Data Analysis**

**Data Acquisition** Resting-state fMRI data were acquired with a Siemens 3T scanner (Siemens Magnetom Trio TIM, Erlangen, Germany). Each scan contains 232 functional volumes (about 5 minutes), collected with an Echo-Planar imaging (EPI) sequence (TR=2 s, TE=30ms, flip angle=75º, matrix size=64×64, FoV= 220×220 mm2, voxel size= 3.4×3.4×3 mm3, Slices=32). Anatomical images were also collected for normalization with a T1-weighted protocol (TR= 1900 ms, TE= 2.52 ms, FA = 9º, matrix = 64×64, FoV = 256×256 mm2, voxel size = 1×1×1 mm3). All subjects were instructed to fixate on the center of the screen, not to think about or concentrate on anything in particular, and to remain as motionless as possible. Head movements were minimized by using a cushioned head fixation device.

**Preprocessing** FMRI data were preprocessed and analyzed using Data Processing Assistant for Resting-State fMRI Advanced Edition (DPARSFA v3.2, <http://rfmri.org/DPARSF>) (Yan, Wang, Zuo, & Zang, 2016) of Data Processing & Analysis of Brain Imaging (DPABI v1.3, http://rfmri.org/DPABI) toolbox, and SPM8 software (http://www.fil.ion.ucl.ac.uk/spm) implemented in Matlab (R2010a, processing MathWorks, Inc., USA). The first ten volumes of each functional time series were discarded to allow for scanner stabilization. The remaining functional images were initially corrected for within-scan acquisition time differences between slices. In order to remove motion related confounds (Power et al., 2014; Power, Schlaggar, & Petersen, 2015; Satterthwaite et al., 2013; Siegel et al., 2016), we performed motion correction using SPM’s realign function to align each individual’s BOLD fMRI data to the mean of the images. During motion correction, head movement was recorded in 6 directions and used to exclude individuals with significant motion (2 mm) and to regress out the effects of motion on BOLD signal. Following motion correction, each individuals’ rsfMRI data were coregistered to their corresponding anatomical image. Each anatomical image was segmented into grey matter, white matter and cerebrospinal fluid probability maps using the ‘New Segment’ function in SPM8 while deriving a deformation field. Following segmentation individuals’ rsfMRI data were transformed to MNI standard space using the deformation field derived during the segmentation step. For all individuals, probability maps for cerebrospinal fluid and white matter were thresholded at p>0.95 to create cerebrospinal fluid (CSF) and white matter (WM) masks respectively. Using these masks, the BOLD time series were extracted from the resting state dataset and the first five principal components were derived. A principal component (PCA) based noise correction (CompCorr) and Friston-24 based GLM model (Friston, Williams, Howard, Frackowiak, & Turner, 1996) was implemented to reduce the effect of physiological noise and motion time series from the BOLD fMRI data using DPABI. The GLM model thus included a total of 34 regressor time-series (5 principal components of white matter, 5 principal components of cerebrospinal fluid, 6 motion parameters, 6 autoregressive motion parameters, and 12 quadratic models of the motion parameters). Finally, normalized functional images were resliced into 3 × 3 × 3 mm3 voxels and spatially smoothed with a Gaussian kernel (8 mm FWHM).

**DMN Identification with Group Independent Component Analysis** The preprocessed data from all the subjects was analyzed with group ICA (GIFT, http://icatb.sourceforge.net/). The optimal number of principal components was set to 30, using the GIFT dimensionality estimation tool. First, data from each subject was reduced using PCA, according to the selected number of components. Second, the data was separated by ICA using the Extended Infomax algorithm. Third, independent components (ICs) and time courses for each subject were back-reconstructed, and the mean spatial maps for each group were transformed to Z-scores for display. The independent component that best matched DMN as previously reported (Greicius & Menon, 2004) was selected, and the corresponding time course DMNICA was used to generate the Hurst exponent estimation.

**DMN Identification with Seed at PCC** Seed based analysis was also used for defining DMN as an attempt to avoid potential selection effects of defining DMN. A single spherical region (radius 10 mm) positioned in the PCC (0, −52, 30) was selected as the seed (Fransson, 2005). Cross-correlation analysis was performed by computing temporal correlation between the mean time course of the seed and BOLD signal intensity of all brain voxels. Correlation coefficients of each voxel were normalized to Z-scores with Fisher's r-to-z transformation, and thus an entire brain Z-score map was created for each subject. The mean signal of all the voxels with a Z-score larger than 3 was regarded as the time course DMNROI, and was input for Hurst exponent estimation.


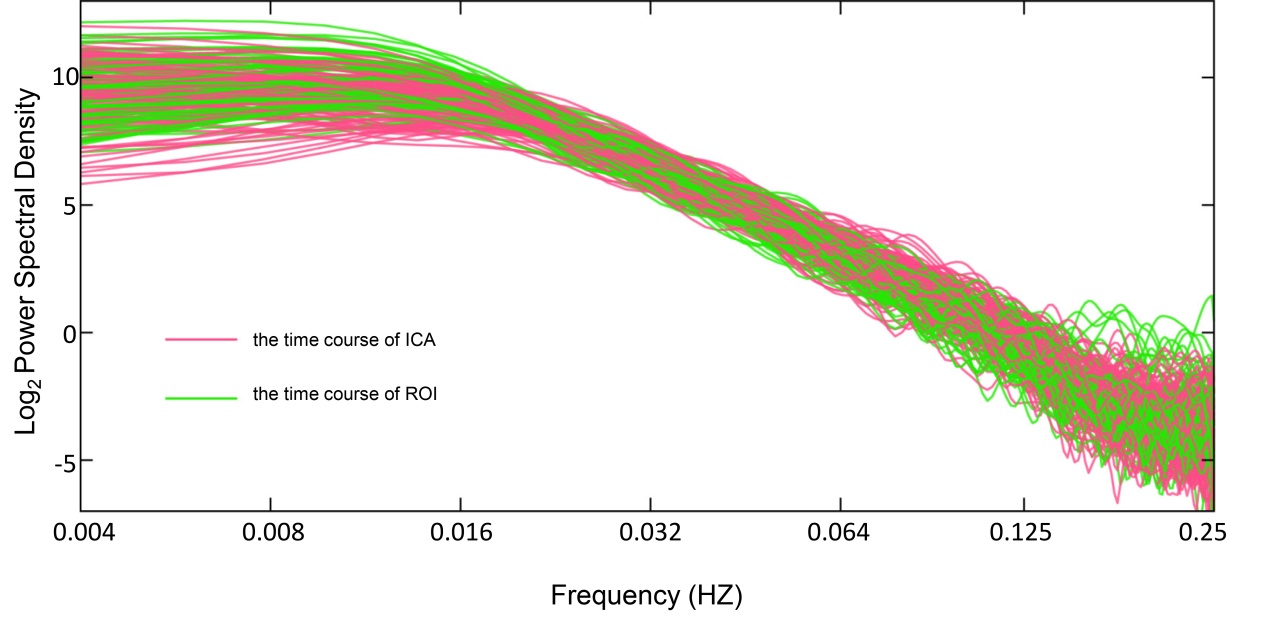


**Fig. 1** The power spectra of all subjects without band-pass filtering from cumulative sum of the time courses of ICA (red) and ROI (green). The scale parameters were chosen to correspond to a frequency range of (0.016, 0.063) Hz. ICA: independent component analysis; ROI: region of interests.

**Hurst exponent** The scale parameters were chosen to correspond to a frequency range of (0.016, 0.063) Hz (Ciuciu, Varoquaux, Abry, Sadaghiani, Kleinschmidt, et al., 2012). For the log–log scale plot of the power spectra, the linear slope (B) is used to calculate the Hurst exponent by the formula H = (B−1) / 2. Further analyses were conducted using Matlab 11.0 (Math Works, Natick, MA), and the Hurst exponent was calculated from DMN_ICA_ and DMN_ROI_ using the WLBMF toolbox (https:// [www.irit.fr/~Herwig.Wendt/](http://www.irit.fr/~Herwig.Wendt/)). For details on our parameters, see Supplement 1. Finally, the Spearman rank correlations were calculated between the Hurst exponent and individuals’ scores on Emotion Regulation Questionnaire. Including the age and gender as covariates led to comparable results.

**fAFLL and ReHo** Other standard measures (fALFF, ReHo) used in resting state MRI analysis were also calculated in this study. ReHo reflects the temporal homogeneity of the regional blood oxygen level-dependent (BOLD) signal and fALFF is the fraction of ALFF in a given frequency band to the ALFF over the entire frequency range detectable in the given signal (Zou et al., 2008). ReHo and fALFF analyses were performed by using the Resting-State fMRI Data Analysis Toolkit (<http://resting-fmri.sourceforge.net>).

**Analysis of DMN subregions** The default network comprises a set of interconnected brain regions, including MPFC, posterior cingulate cortex (PCC) and medial temporal lobe (MTL). Though these subregions, from a point of functional integration, constitutes an organized network during rest (Fox et al., 2005; Greicius, Krasnow, Reiss, & Menon, 2003), increasing evidence has suggested greater heterogeneity within DMN than is commonly appreciated (Roy et al., 2009). Therefore, it is possible that the components of DMN may play different roles in emotion regulation process. To address this issue, we also examined whether the H of DMN at subregion level may mediate individual differences in spontaneous reappraisal and their association with depression. We divided DMN into three key nodes (Andrews-Hanna, 2012) by the ‘‘Free ROI’’ tool (http://freeroi.brainactivityatlas.org).We then extracted the time courses and calculated the Hurst exponent of MPFC, PCC and MTL separately. Finally, H of three key nodes of DMN was involved in the subsequent correlation and mediation analysis.

**RESULTS**

**Spatial patterns of DMN**


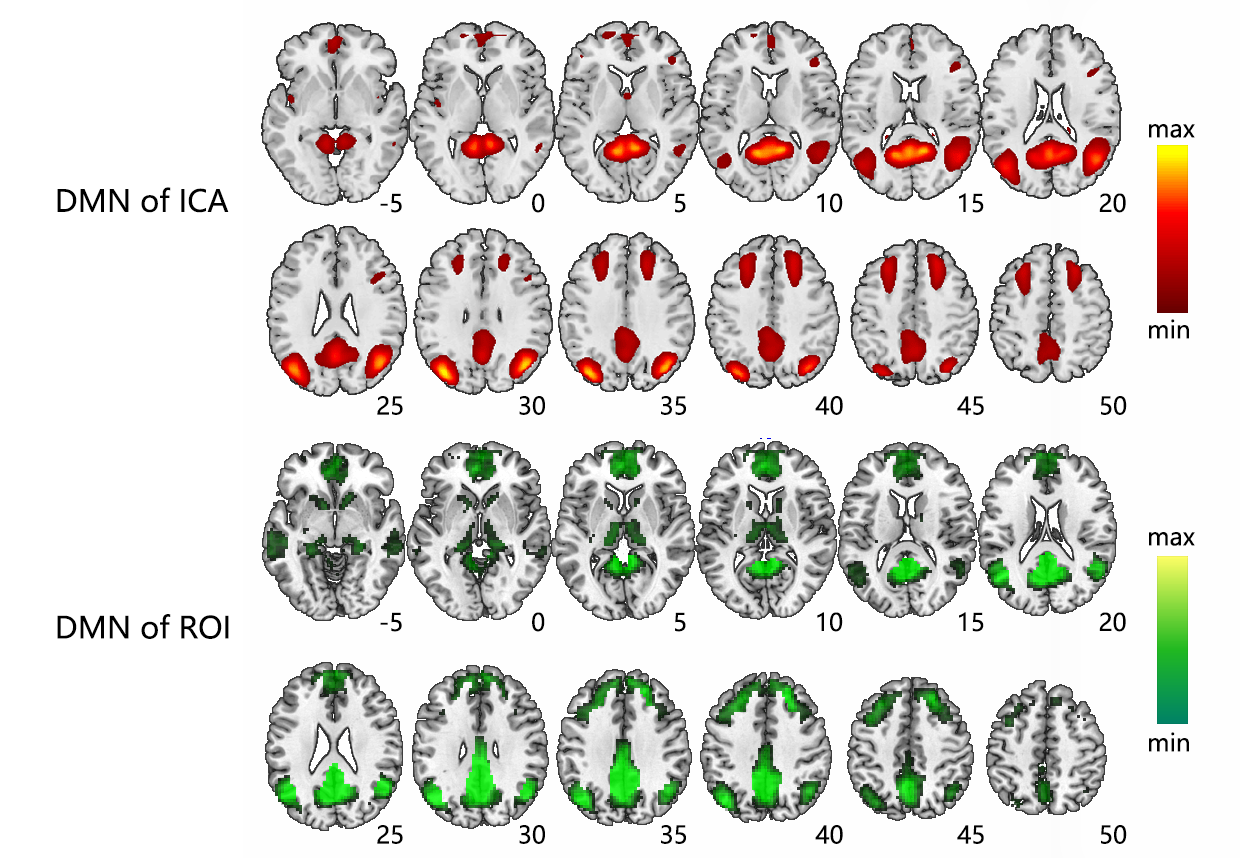


**Fig.2** The spatial patterns of DMN extracted by ICA and ROI. DMN extracted by group ICA (Top) and DMN extracted by ROI seeded in PCC region (Bottom). Axial map was shown to be superimposed on the MNI152 standard space template image. The statistical thresholds of both spatial patterns were p<0.05 (FDR-corrected, 20 adjacent voxels). DMN: default mode network; ICA: independent component analysis; ROI: region of interests; PCC: posterior cingulate/precuneus.

**The correlation between reappraisal and depression scores**

**
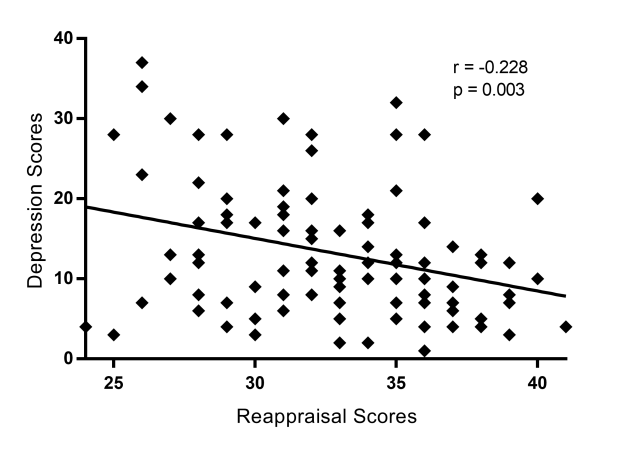
**

**Fig.3** Spearman rank-correlations and scatter-plots displaying the relationship between the scores of reappraisal and depression scores. Significant negative correlation between reappraisal and depression scores, r (105) =−0.288; p < 0.01.

The correlation analysis showed significant negative correlations between reappraisal and depression scores, r (105) =−0.288; p=0.003 (Fig. 3). Consistent with previous studies (Abler et al., 2010; R. C. Martin & Dahlen, 2005; Min et al., 2013), our current result showed that an individual with a higher reappraisal score had a lower depression score.

**Hurst exponents in DMN (including DMN components) and individual differences in reappraisal**

**
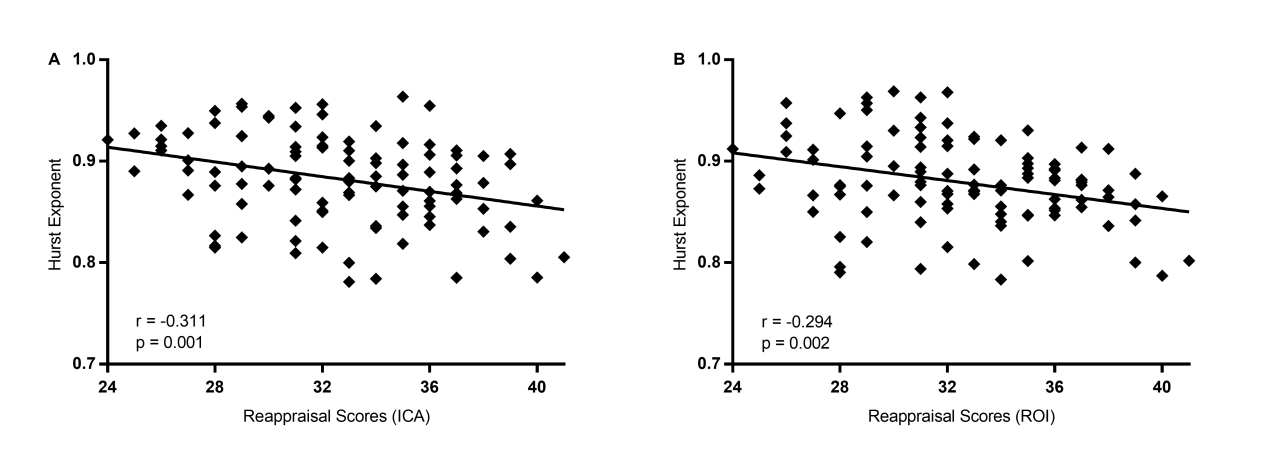
**

**Fig.4** Spearman rank-correlations and scatter-plots displaying the relationship between the scores of reappraisal and the Hurst exponents in DMN_ICA_ (A) and in DMN_ROI_ (B). Significant negative correlation between reappraisal scores and H of DMN_ICA_, r (105) =−0.311; p < 0.01; H of DMN_ROI_, r (105) =−0.294; p < 0.01.

The correlation analysis showed significant negative correlations between reappraisal scores and H of DMN_ICA_, r (105) =−0.311; p=0.001 (Fig. 4A). Thus, an individual with a higher reappraisal score had a lower Hurst value of DMN_ICA_. However, no significant correlations were found between suppression scores and H of DMN_ICA_, r (105) =-0.01, p=0.918. These results indicated that during rest, the temporal complexity of DMN was associated with the use of reappraisal, but not with the use of suppression. Of note, the alternative approach, calculating the correlation between the ERQ scores and H of DMN_ROI_, yielded a similar result that the H of DMN_ROI_ was significantly associated with the use of reappraisal, r (105) =-0.294, p=0.002 (Fig.4B), but not with the use of suppression r (105) =-0.03, p=0.725.

Moreover, given the heterogeneity within DMN, we also examined the relationship between Hurst in key components of DMN and the use of reappraisal. Results showed that the use of reappraisal, but not the use of suppression, was negatively associated with H of PCC, H of MPFC and H of MTL, respectively (see Table 1. For more details on fALFF/ReHo, see Table S1). These findings suggest that the Hurst of both DMN and its components may serve as a specific neuroimaging marker of the spontaneous and uninstructed reappraisal.

**Table 1**

|  | Reappraisal | Suppression |
| --- | --- | --- |
| H of PCC | r = -0.208; p = 0.033 | r = -0.012; p = 0.900 |
| H of MPFC | r = -0.241; p = 0.013 | r = 0.111; p = 0.260 |
| H of MTL | r = -0.210; p = 0.031 | r = 0.065; p = 0.510 |
| fALFF of DMN | r = 0.020; p = 0.840 | r = 0.133; p = 0.175 |
| ReHo of DMN | r = -0.065; p = 0.509 | r = 0.087; p = 0.376 |

**Specificity of findings**

Previous studies demonstrated that extraversion and trait anxiety are correlated with Hurst exponent in DMN (Gentili et al., 2015; Lei et al., 2013) and reappraisal (Martin & Dahlen, 2005; Uliaszek et al., 2010). In order to ensure our findings independent of emotional reactivity (C Gentili et al., 2015) and personality trait (Lei, Zhao, & Chen, 2013), we used a hierarchical regression analysis to test 1) whether extraversion and trait anxiety scores were related to H of DMN and its components, and 2) whether the relationship between reappraisal and H of DMN (including its components) withstood correction for these factors. Regression analyses on H of DMN showed that 14.4% of the variance in the criterion variable was accounted for by the statistical model (F (3, 101) = 5.661; p < 0.001) (See Table 2). Reappraisal captured 9.7% of the variance while the addition of trait anxiety and extraversion to the equation did not result in a significant increment of R^2^. These findings indicated that our primary results were not due to individual differences in emotional reactivity or personality trait, and specific to reappraisal.

Other standard measures (fALFF, ReHo) used in resting state MRI measures were also calculated using these indexes. No significant correlations were found between fALFF/ReHo of DMN and reappraisal scores (see Table 1). Therefore, the association between DMN and spontaneous reappraisal is specific to the Hurst index of this network, as other standard measures (fALFF, ReHo) cannot reflect the relationship between DMN activity and spontaneous reappraisal. Previous studies have indicated that fALFF and ReHo help to reveal the complexity of the brain function, by reflecting the intensity and homogeneity of regional spontaneous brain activity, respectively (Zang et al., 2004; Zou et al., 2008). However, these indicators cannot reflect the temporal dynamic property of spontaneous brain activity. Therefore, our current results suggested that temporal scale of spontaneous brain signals may play a more important role in emotion regulation process and depression.

**Table 2** **Regression coefficients (R^2^, △R^2^) and statistical results of** **hierarchical linear regression analyses on H of DMN with respect to the influence of reappraisal, trait anxiety and extraversion are shown.**

| Dependent Variables Step | | H of DMN | | | |
| --- | --- | --- | --- | --- | --- |
|  |  | Beta R^2^ △R^2^ | | | P< |
| reappraisal | 0.097 0.001 | | | | |
|  | reappraisal alone | -0.312 |  | 0.097 | 0.001 |
| reappraisal, TA | 0.114 0.002 | | | | |
|  | reappraisal added first | -0.275 |  | 0.097 | 0.005 |
|  | TA added second | 0.135 |  | 0.017 | 0.166 |
| reappraisal, TA, extraversion | 0.144 0.001 | | | | |
|  | reappraisal added first | -0.263 |  | 0.097 | 0.007 |
|  | TA added second | 0.136 |  | 0.017 | 0.159 |
|  | extraversion added third | -0.172 |  | 0.030 | 0.065 |

TA: trait anxiety. Probability values are two-tailed. R^2^ illustrates the regression model, whereas △R^2^ illustrates the improvement of the regression model when additional independent variables are considered.

**H of DMN mediates the association of reappraisal with depression**

The mediation analysis is based on a standard three-variable path model and with a bootstrap test for the statistical significance of the indirect effect, as diagrammed in Fig 5. As illustrated in Figure 5, the results showed that the indirect effects of the use of reappraisal on the subjective depression scores were significant (H of DMN_ICA_: a*b = -0.0718, p< 0.05; H of DMN_ROI_: a*b = -0.1023, p< 0.05). But further mediation analysis results of three subregions of DMN showed that only H of MPFC mediates the association between the use of reappraisal and depression. For details on our mediation analysis, see Supplement 1. These results, shown in Table 3, indicate that resting-state temporal complexity in DMN explains a part of the reappraisal and depression association.


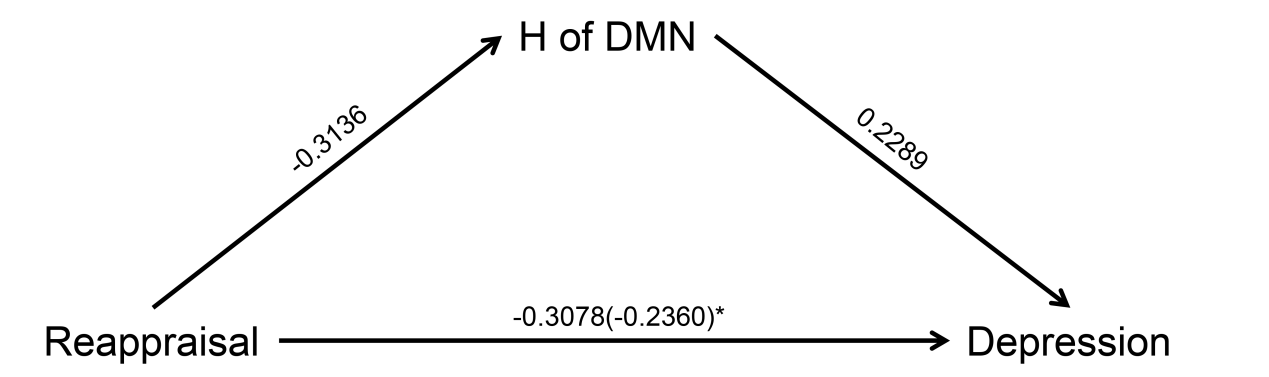


**Fig.5** The relationship between reappraisal and depression scores was mediated by H of DMN. The lines are labeled with path coefficients, and standard errors are shown in parentheses. The direct path between spontaneous reappraisal and depression is calculated controlling for H. Indirect path a = -0.31136, indirect path b = 0.2289, total relationship c = -0.3078 and direct path c’ = -0.2360. The values in parentheses indicate the strength of the path prior to the inclusion of the mediating variable, *p < 0 .05.

**Table 3** **Mediation effects of H of DMN in the prediction of Depression (N= 105).**

| Mediator | Depression | |
| --- | --- | --- |
|  | Point Estimate (a*b) | Bootstrapping BC 95% CI |
| H of DMN_ICA_ | -0.0718 | [-0.1651, -0.0112] |
| H of DMN_ROI_ | -0.0691 | [-0.1706, -0.0105] |
| H of PCC | -0.0180 | [-0.0818, 0.0306] |
| H of MPFC | -0.0788 | [-0.1897, -0.0175] |
| H of MTL | 0.0218 | [-0.0147, 0.0871] |

**Note:** The mediation effects of the use of reappraisal on subjective depression were bootstrapped using 5000 samples. Ninety-five percent bias-corrected confidence intervals for all indirect effects and contrasts were generated.

**DISCUSSION**

Most literature on DMN-reappraisal association to date focused on the functional connectivity between brain regions. The present fMRI study explored the temporal dynamics of DMN during resting-state and examined the association between the Hurst exponent of DMN and the ERQ scores. Our findings indicate that more use of spontaneous reappraisal predicts less memory of resting-state neural signals (i.e. Hurst exponents closer to 0.5) in DMN, regardless of whether the Hurst exponent was extracted by ROI or ICA. Importantly, further mediation analysis indicated that the temporal dynamics of DMN (particularly the MPFC) measured by Hurst exponent mediate the association between spontaneous reappraisal and depression. Our findings suggest that temporal scale of spontaneous brain signals may play an important role in emotion regulation process and its association with depression.

In the present study, we found that a higher Hurst exponent of DMN corresponds to a higher risk of depression. This appears inconsistent with previous findings that DMN exhibited decreased Hurst value in major depressive disorder as compared with healthy controls (Wei et al., 2015; Wei et al., 2013). We consider that two reasons may contribute to this difference. On the one hand, as stated before, different algorithms were used to represent Hurst exponent in the above and the current studies, driven by different research purposes. We used original Hurst value that ranges continuously from 0 to 1, while Wei and colleagues used weight vectors in support vector machine approach, to represent the H. On the other hand, individual differences in healthy population, as involved in the current study, may be reflected by distinct DMN temporal dynamic profiles from those in clinical population. Specifically, Wei and colleagues considered that the decreased H may suggest irregular regional oscillation originated from persistent negative thoughts in MDD. However, previous studies demonstrated that there are different behavior and brain mechanism between MDD and healthy people (Kaiser et al., 2016; Tozzi et al., 2017; Wang et al., 2015). For instance, Kaiser et al. found that MDD with ruminative thinking have abnormal patterns of fluctuating communication among brain systems compared with healthy people (Kaiser et al., 2016). In this regard, healthy population, who have no symptoms of persistent negative thoughts and ruminative coping (D’Avanzato, Joormann, Siemer, & Gotlib, 2013; Zetsche, D'Avanzato, & Joormann, 2012), may have distinct individual difference profiles in the temporal dynamics of DMN. That is, the implication of individual differences in Hurst exponent of DMN should be considered in the context of different populations. The relationship between emotion regulation strategy and Hurst exponent of DMN in depressed patients should be examined in future studies.

The Hurst exponent is a useful measure to characterize different physiological states, as shown multiple times (Berthouze, James, & Farmer, 2010; Bojic & Vuckovic, 2010; Churchill et al., 2016; Ciuciu, Varoquaux, Abry, Sadaghiani, & Kleinschmidt, 2012; Kantelhardt, Tismer, Gans, Schumann, & Penzel, 2015; Tagliazucchi et al., 2013). According to previous evidences, a smaller value of H suggests the brain network is more efficient in online information processing and less in long-range memory (Yang & Tsai, 2013). However, a recent study suggests that the stationary fractional Gaussian noise (fGn) process is not sufficient to describe neural data (Von, Laufs, & Tagliazucchi, 2018). So the interpretation of memory effects in real-world signals may benefit from information-theoretical analyses, in addition to Hurst exponent estimation. As a result, the interpretation that the long-range memory can be predicted by Hurst exponents correctly should be more cautious.

Resting state studies of spontaneous fluctuations in fMRI signals have demonstrated huge potential in mapping the brain’s intrinsic functional features (Krüger & Glover, 2001; Yan et al., 2010). Ciuciu et al. found that spontaneous brain activity exhibits scale-free dynamics, suggesting the temporal complexity and fractal-like of the resting-state BOLD signal (Ciuciu et al., 2012). In previous study, researchers quantified the temporal complexity of rs-fMRI based on H, because H can reflect the property of scale-free dynamics via describing the self-similarity of time courses (Maxim et al., 2005; Park, Lazar, Ahn, & Sornborger, 2010). The complexity of resting-state BOLD signals could provide some evidence of dynamics of intrinsic brain activity (Yang et al., 2013). Wink et al. utilized H to quantify fractal complexity and describe pathological and physiological features, then found that normal aging is accompanied by a loss of complexity (decreased H) in bilateral hippocampus. Therefore, in the present study, the negative correlation between reappraisal and H of DMN may suggest that participant with a higher reappraisal score (a lower H value) is associated with increase of complexity in the DMN. Recently, Dong and colleagues used the H exponent to explore fractal complexity of the rs-fMRI signal in the human brain across the adult lifespan (Dong et al., 2018). And they found a significant positive correlation between the mean H of whole brain gray matter and the age of all subjects, suggesting that H increases with age. That is, complexity of BOLD activity is reduced with age. Further, their results showed that healthy aging is accompanied by reduced complexity (increased H) in frontal and parietal lobes and by increased complexity (decreased H) in insula, limbic, and temporal lobes. They speculate that age-related increase of complexity in insula is due to that insula is critical for emotional feeling (Gasquoine, 2014), and with aging, the adult’s ability to regulate emotion remains stable and improves in some aspects (Nashiro, Sakaki, & Mather, 2012). Previous studies have suggested that the DMN is critical for self-referential processing, affective cognition, and emotion regulation (Andrewshanna, Reidler, Sepulcre, Poulin, & Buckner, 2010; Buckner, Andrews-Hanna, & Schacter, 2008). Therefore, it is reasonable that increase of complexity (decreased H) in the DMN is associated with a higher reappraisal score.

Prior studies have indicated a close the association between information processing efficiency in the brain and one’s cognitive flexibility (Blackwell, Cepeda, & Munakata, 2009; Ragozzino et al., 2012; Wingenfeld, Wolf, Krieg, & Lautenbacher, 2011). In this regard, cognitive flexibility might be another interpretation for our results. Cognitive flexibility is defined as—the ability to adjust one’s emotional, cognitive, and behavioral responding to a situation based on new information (Johnco et al., 2014; M. M. Martin & Rubin, 1995). Cognitive flexibility predicts effective reappraisal of emotion situation (Malooly & Ashley, 2012), and is closely linked to rumination (i.e., failure to shift thoughts away from a past threat; (J Joormann, Levens, & Gotlib, 2011; Nolen-Hoeksema, 2000)) and worry (i.e., failure to shift thoughts away from future threat; (Lee & Orsillo, 2013)). It has been reported that cognitive inflexibility is associated with difficulty in adjusting cognition, attitude and behavior despite the input of new information (Armanda, Hamtiaux, Claude, & Houssemand, 2012). And previous studies have demonstrated that cognitive flexibility is associated with depression and depressed patients have impaired cognitive flexibility (Jutta Joormann & Siemer, 2011; F. C. Murphy, Michael, & Sahakian, 2012). That is, similar to the Hurst of DMN that mediates the association between reappraisal and depression, cognitive flexibility also predicts both reappraisal and depression. On the other hand, the Hurst exponent reflects the temporal complexity of neural activity patterns, which is analogous to the continuation of the established cognitive schema over time, as reflected by cognitive inflexibility. Based on these considerations, we posit that a higher Hurst may correspond to greater cognitive inflexibility. That is, people with a higher Hurst value (worse online information processing), are less effective in reappraisal and at higher risk of depression, most likely as a result of cognitive inflexibility. However, caution should be exercised during inferring the Hurst of DMN to cognitive flexibility, as this variable was not directly assessed in the current study. This potential association needs to be addressed in future studies.

The mediation analysis showed that the relationship between spontaneous reappraisal use and depression is mediated by H from DMN, particularly the MPFC. Several previous studies have shown that MPFC is generally involved in cognitive control, and has important relation with successful reappraisal during emotion regulation (Buhle et al., 2014; Etkin, Egner, Peraza, Kandel, & Hirsch, 2006; Goldin, Mcrae, Ramel, & Gross, 2008; Ridderinkhof, Ullsperger, Crone, & Nieuwenhuis, 2004). Goldin et al. (2008) suggest that MPFC regions play a crucial role in down-regulating neural responses of emotion-generating-regions to negative stimuli, like amygdala and insula. Many studies have suggested that the other two subregions in DMN are involved in both memory and emotion. There is considerable evidence that the MTL has functions related to episodic or remote autobiographical memories (Aggleton, 2012; Buchanan, Tranel, & Adolphs, 2005; Phelps, 2004; Simons & Spiers, 2003) and the PCC shows increased activity when individuals retrieve autobiographical memories or plan for the future (Addis, Wong, & Schacter, 2007; Leech & Sharp, 2013; Mantani, Okamoto, Shirao, Okada, & Yamawaki, 2005; Mason et al., 2007). Together with the function of three subregions in DMN mentioned in the previous paragraph, our results indicate that people who use reappraisal more (lower Hurst value) tend to have a greater capability of online information processing, which facilitate the cognitive control of MPFC. What’s more, a shorter long-range memory in PCC or MTL can help people to ruminate less but more engage in online cognitive activity, which facilitate successful reappraisal.

However, further mediation analysis showed that only the temporal complexity of MPFC mediate the relationship between reappraisal use and depression. One possible explanation is that MPFC is involved in both cognitive control and self-referential processing. MPFC is critical in internal, self-referential processing (Northoff et al., 2006), and has been suggested to play an important role in self-referential processing in major depression (Lemogne, Delaveau, Freton, Guionnet, & Fossati, 2012; Lemogne et al., 2010; Mitchell, Banaji, & Macrae, 2005). Lemogne et al. (2012) has provided a compelling evidence for the role of an increased MPFC activity in the depressive self-focus which is associated with acute depressive states and with an increased risk of depressive relapse through ruminative processes. Additionally, previous studies have shown that successful performance of cognitive flexibility depends on the normal structure and function of MPFC (Dalley, Cardinal, & Robbins, 2004; Hang et al., 2016). For instance, both lesions in MPFC and alterations of the level of MPFC dopamine have been found to induce disturbances in cognitive flexibility (Hernandez et al., 2016; Logue & Gould, 2014). Hurst exponent has been proposed as a measure of online information-processing efficiency: lower Hurst values are related to lower temporal redundancy and more freedom to vary (cognitive flexibility) (B. J. He, 2011). Therefore, we suggest that differences of the temporal complexity in spontaneous MPFC activity may represent a possible neurobiological correlate of cognitive flexibility. Together, we suggest a loss of complexity in MPFC may reflect a decreased ability of cognitive control, more rumination on self-referential memories and cognitive inflexibility, which causes vulnerability to depression.

**Clinical Implications**

There is an increasing consensus that dysfunctional emotion regulation is a core feature of major psychiatric illnesses like depression or anxiety disorders (Abler et al., 2010; R. C. Martin & Dahlen, 2005; Min et al., 2013). Decreased use of reappraisal in particular, as indexed by the ERQ, is associated with increased depressive symptoms (Gross & John, 2003). In addition, a series of studies have shown that dysfunction of the default-mode network is associated with depression (Johnstone et al., 2007; Sheline et al., 2009; Shi et al., 2015; Wei et al., 2015). Our results suggest the temporal complexity in DMN plays a crucial mediating role in the relationship between the use of reappraisal and depression. This suggests that people who use reappraisal less (higher Hurst value) tend to have a loss of complexity of neural signals in DMN, implying reduced cognitive flexibility (and difficulty in adapting to environmental changes). Those people are more likely to ruminate in the negative context, which causes them to fall into stronger depression. The present study, therefore, offers a consideration on depression treatment by changing the temporal complexity in DMN, for example, by the reappraisal training.

**Limitations and Future Directions**

The study has five limitations that necessitate future investigation and research. Firstly, only rest state data were studied. Therefore, it is possible that the results from this study cannot be generalized to task activity studies. Future studies should combine with emotion regulation task to further study this association. Secondly, although the association between H of DMN and the use of reappraisal has been examined by different methods and appear to be robust, these findings are limited only to the index of H of DMN. Therefore, future studies should apply multiple approaches to delineate brain network dynamics like sliding window methods (Kaiser et al., 2016), co-activation pattern (CAP) analysis (Chen, Chang, Greicius, & Glover, 2015) and multi-layered dynamic analysis (Raz et al., 2012) to further confirm the observed associations in follow-up studies. Third, only healthy young participants were studied, and it is therefore unknown whether our results are generalizable to all age group. Because age differences are often observed in studies of emotion regulation (Martins, Sheppes, Gross, & Mather, 2016; Scheibe, Sheppes, & Staudinger, 2015), it will be important to examine whether the association between emotion regulation tendencies and age differences is exist. Forth, MDD patients is not involved in the current study. Thus, individual differences in temporal dynamics of DMN in MDD patients and its association with emotion regulation tendencies should be examined in future studies. Finally, Recent study found that the mutual information function of neurophysiological data behaves differently from fractional Gaussian noise (fGn), and the H phenomenon is a sufficient condition to prove long-range memory only in the stationary fGn process (Von, Laufs, & Tagliazucchi, 2018). Thus, the interpretation that the long-range memory can be predicted by Hurst exponents correctly should be more cautious, and future study should use the time-lagged mutual information function (a novel and effective tool to assess long-range dependence in finite length empirical data) as a complementary method to measure memory effects.

**Conclusion**

In conclusion, the present study provides first evidence for a negative association between the use of cognitive reappraisal and the temporal dependence of DMN during resting-state. Specifically, individual with a higher reappraisal score has a lower Hurst value of DMN as shown by the resting state functional MRI. More importantly, our mediation results suggest the temporal complexity of DMN play an important role in the relationship between spontaneous reappraisal and depression, which shed light on depression intervention in the field of clinical practice. These findings suggest that H of DMN at rest may serve as a neuroimaging marker of one’s spontaneous, uninstructed reappraisal and its association with depression.

**ACKNOWLEDGMENTS AND DISCLOSURES**

This scientific work was supported by National Natural Science Foundation of China (No. 30870668, 81273674) to Jiajin Yuan.

All authors report no biomedical financial interests or potential conflicts of interest.

**REFERENCES**

Abler, B., Hofer, C., Walter, H., Erk, S., Hoffmann, H., Traue, H. C., & Kessler, H. (2010). Habitual emotion regulation strategies and depressive symptoms in healthy subjects predict fMRI brain activation patterns related to major depression. *Psychiatry Res, 183*(2), 105-113.

Addis, D. R., Wong, A. T., & Schacter, D. L. (2007). Remembering the past and imagining the future: common and distinct neural substrates during event construction and elaboration. *Neuropsychologia, 45*(7), 1363-1377.

Aggleton, J. P. (2012). Multiple anatomical systems embedded within the primate medial temporal lobe: implications for hippocampal function. *Neuroscience & Biobehavioral Reviews, 36*(7), 1579-1596.

Andrews-Hanna, J. R. (2012). The brain’s default network and its adaptive role in internal mentation. *The Neuroscientist, 18*(3), 251-270.

Andrewshanna, J. R., Reidler, J. S., Sepulcre, J., Poulin, R., & Buckner, R. L. (2010). Functional-Anatomic Fractionation of the Brain's Default Network. *Neuron, 65*(4), 550-562.

Armanda, Hamtiaux, Claude, & Houssemand. (2012). Adaptability, Cognitive Flexibility, Personal Need for Structure, and Rigidity. *心理学研究:英文版*(10), 563-585.

Beauregard, M., Paquette, V., & Lévesque, J. (2006). Dysfunction in the neural circuitry of emotional self-regulation in major depressive disorder. *Neuroreport, 17*(8), 843.

Beck, A. T., Ward, C. H., Mendelson, M., Mock, J., & Erbaugh, J. (1961). An inventory for measuring depression. *Archives of General Psychiatry, 4*(6), 561-571.

Berthouze, L., James, L. M., & Farmer, S. F. (2010). Human EEG shows long-range temporal correlations of oscillation amplitude in Theta, Alpha and Beta bands across a wide age range. *Clinical Neurophysiology Official Journal of the International Federation of Clinical Neurophysiology, 121*(8), 1187.

Blackwell, K. A., Cepeda, N. J., & Munakata, Y. (2009). When simple things are meaningful: Working memory strength predicts children’s cognitive flexibility. *Journal of Experimental Child Psychology, 103*(2), 241-249.

Bojic, T., & Vuckovic, A. A. (2010). Modeling EEG fractal dimension changes in wake and drowsy states in humans--a preliminary study. *Journal of Theoretical Biology, 262*(2), 214-222.

Buchanan, T. W., Tranel, D., & Adolphs, R. (2005). Emotional autobiographical memories in amnesic patients with medial temporal lobe damage. *Journal of Neuroscience, 25*(12), 3151-3160.

Buckner, R. L., Andrews-Hanna, J. R., & Schacter, D. L. (2008). The brain's default network: anatomy, function, and relevance to disease. Ann N Y Acad Sci, 1124, 1-38. doi:10.1196/annals.1440.011

Buhle, J. T., Silvers, J. A., Wager, T. D., Lopez, R., Onyemekwu, C., Kober, H., . . . Ochsner, K. N. (2014). Cognitive reappraisal of emotion: a meta-analysis of human neuroimaging studies. *Cereb Cortex, 24*(11), 2981-2990. doi:10.1093/cercor/bht154

Chao, W., Shen, Z., Huang, P., Yu, H., Wei, Q., Guan, X., . . . Zhang, M. (2017). Altered spontaneous brain activity in chronic smokers revealed by fractional ramplitude of low-frequency fluctuation analysis: a preliminary study. *Sci Rep, 7*(1).

Chen, J. E., Chang, C., Greicius, M. D., & Glover, G. H. (2015). Introducing co-activation pattern metrics to quantify spontaneous brain network dynamics. *Neuroimage, 111*, 476-488.

Churchill, N. W., Spring, R., Grady, C., Cimprich, B., Askren, M. K., Reuterlorenz, P. A., . . . Berman, M. G. (2016). The suppression of scale-free fMRI brain dynamics across three different sources of effort: aging, task novelty and task difficulty. Sci Rep, 6, 30895.

Ciuciu, P., Varoquaux, G., Abry, P., Sadaghiani, S., Kleinschmidt, & A. (2012). Scale-Free and Multifractal Time Dynamics of fMRI Signals during Rest and Task. *Frontiers in Physiology, 3*, : 186.

D’Avanzato, C., Joormann, J., Siemer, M., & Gotlib, I. H. (2013). Emotion Regulation in Depression and Anxiety: Examining Diagnostic Specificity and Stability of Strategy Use. *Cognitive Therapy and Research, 37*(5), 968-980. doi:10.1007/s10608-013-9537-0

Dalley, J. W., Cardinal, R. N., & Robbins, T. W. (2004). Prefrontal executive and cognitive functions in rodents: neural and neurochemical substrates. *Neurosci Biobehav Rev, 28*(7), 771-784.

Deco, G., Jirsa, V. K., & Mcintosh, A. R. (2011). Emerging concepts for the dynamical organization of resting-state activity in the brain. *Nature Reviews Neuroscience, 12*(1), 43-56.

Denier, N., Schmidt, A., Gerber, H., Vogel, M., Huber, C. G., Lang, U. E., . . . Walter, M. (2015). Abnormal functional integration of thalamic low frequency oscillation in the BOLD signal after acute heroin treatment. *Human brain mapping, 36*(12), 5287-5300.

Dillon, D. G., & Pizzagalli, D. A. (2013). Evidence of successful modulation of brain activation and subjective experience during reappraisal of negative emotion in unmedicated depression. *Psychiatry Res, 212*(2), 99-107.

Disner, S. G., Beevers, C. G., Haigh, E. A., & Beck, A. T. (2011). Neural mechanisms of the cognitive model of depression. *Nat Rev Neurosci, 12*(8), 467-477. doi:10.1038/nrn3027

Dong, J., Jing, B., Ma, X., Liu, H., Xiao, M., & Li, H. (2018). Hurst Exponent Analysis of Resting-State fMRI Signal Complexity across the Adult Lifespan. Frontiers in Neuroscience, 12, 34.

Egloff, B., Schmukle, S. C., Burns, L. R., & Schwerdtfeger, A. (2006). Spontaneous emotion regulation during evaluated speaking tasks: associations with negative affect, anxiety expression, memory, and physiological responding. *Emotion, 6*(3), 356.

Ehring, T., Tuschen-Caffier, B., Schnulle, J., Fischer, S., & Gross, J. J. (2010). Emotion regulation and vulnerability to depression: spontaneous versus instructed use of emotion suppression and reappraisal. *Emotion, 10*(4), 563-572. doi:10.1037/a0019010

Ertl, M., Hildebrandt, M., Ourina, K., Leicht, G., & Mulert, C. (2013). Emotion regulation by cognitive reappraisal—the role of frontal theta oscillations. *Neuroimage, 81*, 412-421.

Etkin, A., Egner, T., Peraza, D. M., Kandel, E. R., & Hirsch, J. (2006). Resolving emotional conflict: a role for the rostral anterior cingulate cortex in modulating activity in the amygdala. *Neuron, 51*(6), 871-882.

Expert, P., Lambiotte, R., Chialvo, D. R., Christensen, K., Jensen, H. J., Sharp, D. J., & Turkheimer, F. (2011). Self-similar correlation function in brain resting-state functional magnetic resonance imaging. Journal of the Royal Society Interface, 8(57), 472.

Fox, M. D., Snyder, A. Z., Vincent, J. L., Corbetta, M., Van Essen, D. C., & Raichle, M. E. (2005). The human brain is intrinsically organized into dynamic, anticorrelated functional networks. *Proc Natl Acad Sci U S A, 102*(27), 9673-9678.

Fox, M. D., Snyder, A. Z., Vincent, J. L., & Raichle, M. E. (2007). Intrinsic fluctuations within cortical systems account for intertrial variability in human behavior. *Neuron, 56*(1), 171-184. doi:10.1016/j.neuron.2007.08.023

Fransson, P. (2005). Spontaneous low-frequency BOLD signal fluctuations: an fMRI investigation of the resting-state default mode of brain function hypothesis. *Hum Brain Mapp, 26*(1), 15-29. doi:10.1002/hbm.20113

Friston, K. J., Williams, S., Howard, R., Frackowiak, R. S., & Turner, R. (1996). Movement‐related effects in fMRI time‐series. *Magnetic resonance in medicine, 35*(3), 346-355.

Gasquoine, P. G. (2014). Contributions of the Insula to Cognition and Emotion. Neuropsychology Review, 24(2), 77.

Gentili, C., Vanello, N., Cristea, I., David, D., Ricciardi, E., & Pietrini, P. (2015). Proneness to social anxiety modulates neural complexity in the absence of exposure: A resting state fMRI study using Hurst exponent. *Psychiatry Research Neuroimaging, 232*(2), 135-144.

Goldin, P. R., Mcrae, K., Ramel, W., & Gross, J. J. (2008). The neural bases of emotion regulation: reappraisal and suppression of negative emotion. *Biological Psychiatry, 63*(6), 577.

Grav, S., Stordal, E., Romild, U. K., & Hellzen, O. (2012). The relationship among neuroticism, extraversion, and depression in the HUNT Study: in relation to age and gender. *Issues Ment Health Nurs, 33*(11), 777-785.

Greicius, M. D., Krasnow, B., Reiss, A. L., & Menon, V. (2003). Functional Connectivity in the Resting Brain: A Network Analysis of the Default Mode Hypothesis.

Greicius, M. D., & Menon, V. (2004). Default-mode activity during a passive sensory task: uncoupled from deactivation but impacting activation. *Journal of Cognitive Neuroscience, 16*(9), 1484-1492.

Gross, J. J., & John, O. P. (2003). Individual differences in two emotion regulation processes: Implications for affect, relationships, and well-being. *Journal of Personality and Social Psychology, 85*(2), 348-362. doi:10.1037/0022-3514.85.2.348

Hamilton, J. P., Furman, D. J., Chang, C., Thomason, M. E., Dennis, E., & Gotlib, I. H. (2011). Default-mode and task-positive network activity in major depressive disorder: implications for adaptive and maladaptive rumination. *Biol Psychiatry, 70*(4), 327-333. doi:10.1016/j.biopsych.2011.02.003

Hang, X., Yu, Z., Fan, Z., Yuan, S., Feng, S., & Wang, W. (2016). Effects of Duloxetine Treatment on Cognitive Flexibility and BDNF Expression in the mPFC of Adult Male Mice Exposed to Social Stress during Adolescence. *Frontiers in Molecular Neuroscience, 9*.

He, B. J. (2014). Scale-free brain activity: past, present, and future. Trends in Cognitive Sciences, 18(9), 480-487.

He, B. J. (2011). Scale-free properties of the functional magnetic resonance imaging signal during rest and task. *Journal of Neuroscience the Official Journal of the Society for Neuroscience, 31*(39), 13786-13795.

He, B. J., Zempel, J. M., Snyder, A. Z., & Raichle, M. E. (2010). The Temporal Structures and Functional Significance of Scale-free Brain Activity. *Neuron, 66*(3), 353-369.

Hernandez, A. R., Reasor, J. E., Truckenbrod, L. M., Lubke, K. N., Johnson, S. A., Bizon, J. L., . . . Burke, S. N. (2016). Medial Prefrontal-Perirhinal Cortical Communication is Necessary for Flexible Response Selection. *Neurobiology of Learning & Memory, 137*.

Janssonfröjmark, M., & Lindblom, K. (2008). A bidirectional relationship between anxiety and depression, and insomnia? A prospective study in the general population. *Journal of Psychosomatic Research, 64*(4), 443-449.

Jiang, L., & Zuo, X. N. (2016). Regional Homogeneity. *Neuroscientist, 22*(5), 486-505.

Johnco, C., Wuthrich, V. M., & Rapee, R. M. (2014). The influence of cognitive flexibility on treatment outcome and cognitive restructuring skill acquisition during cognitive behavioural treatment for anxiety and depression in older adults: Results of a pilot study. *Behav Res Ther, 57*(1), 55.

Johnstone, T., van Reekum, C. M., Urry, H. L., Kalin, N. H., & Davidson, R. J. (2007). Failure to regulate: counterproductive recruitment of top-down prefrontal-subcortical circuitry in major depression. *J Neurosci, 27*(33), 8877-8884. doi:10.1523/JNEUROSCI.2063-07.2007

Joormann, J., & Gotlib, I. H. (2010). Emotion regulation in depression: Relation to cognitive inhibition. *Cogn Emot, 24*(2), 281-298. doi:10.1080/02699930903407948

Joormann, J., Levens, S. M., & Gotlib, I. H. (2011). Sticky thoughts: depression and rumination are associated with difficulties manipulating emotional material in working memory. *Psychological Science, 22*(8), 979-983.

Joormann, J., & Siemer, M. (2011). Affective Processing and Emotion Regulation in Dysphoria and Depression: Cognitive Biases and Deficits in Cognitive Control. *Social & Personality Psychology Compass, 5*(1), 13–28.

Kaiser, R. H., Whitfieldgabrieli, S., Dillon, D. G., Goer, F., Beltzer, M., Minkel, J., . . . Pizzagalli, D. A. (2016). Dynamic Resting-State Functional Connectivity in Major Depression. *Neuropsychopharmacology, 41*(7), 1822-1830.

Kantelhardt, J. W., Tismer, S., Gans, F., Schumann, A. Y., & Penzel, T. (2015). Scaling behavior of EEG amplitude and frequency time series across sleep stages. Epl, 112(1), 18001.

Koval, P., Butler, E. A., Hollenstein, T., Lanteigne, D., & Kuppens, P. (2015). Emotion regulation and the temporal dynamics of emotions: Effects of cognitive reappraisal and expressive suppression on emotional inertia. *Cogn Emot, 29*(5), 831-851. doi:10.1080/02699931.2014.948388

Krüger, G., & Glover, G. H. (2001). Physiological noise in oxygenation-sensitive magnetic resonance imaging. Magnetic Resonance in Medicine, 46(4), 631.

Lai, M. C., Lombardo, M. V., Chakrabarti, B., Sadek, S. A., Pasco, G., Wheelwright, S. J., . . . Suckling, J. (2010). A shift to randomness of brain oscillations in people with autism. *Biological Psychiatry, 68*(12), 1092-1099.

Lau, W. K., Leung, M.-K., Chan, C. C., Wong, S. S., & Lee, T. M. (2015). Can the neural–cortisol association be moderated by experience-induced changes in awareness?

Lee, J. K., & Orsillo, S. M. (2013). Investigating cognitive flexibility as a potential mechanism of mindfulness in Generalized Anxiety Disorder. *Journal of Behavior Therapy & Experimental Psychiatry, 45*(1), 208-216.

Leech, R., & Sharp, D. J. (2013). The role of the posterior cingulate cortex in cognition and disease. *Brain, 137*(1), 12-32.

Lei, X., Zhao, Z., & Chen, H. (2013). Extraversion is encoded by scale-free dynamics of default mode network. *Neuroimage, 74*(10), 52-57.

Lemogne, C., Delaveau, P., Freton, M., Guionnet, S., & Fossati, P. (2012). Medial prefrontal cortex and the self in major depression. *J Affect Disord, 136*(1), e1-e11.

Lemogne, C., Mayberg, H., Bergouignan, L., Volle, E., Delaveau, P., Lehéricy, S., . . . Fossati, P. (2010). Self-referential processing and the prefrontal cortex over the course of depression: a pilot study. *J Affect Disord, 124*(1), 196-201.

Liao, W., Xu, Q., Mantini, D., Ding, J., Machado-de-Sousa, J. P., Hallak, J. E., . . . Zhang, W. (2011). Altered gray matter morphometry and resting-state functional and structural connectivity in social anxiety disorder. *Brain Res, 1388*, 167-177.

Logue, S. F., & Gould, T. J. (2014). The Neural and Genetic Basis of Executive Function: Attention, Cognitive Flexibility, and Response Inhibition. *Pharmacology Biochemistry & Behavior, 123*, 45-54.

Malooly, & Ashley, M. (2012). The Role of Affective Flexibility and Cognitive Flexibility in Effective Antecedent-Focused and Online Reappraisal.

Mantani, T., Okamoto, Y., Shirao, N., Okada, G., & Yamawaki, S. (2005). Reduced activation of posterior cingulate cortex during imagery in subjects with high degrees of alexithymia: a functional magnetic resonance imaging study. *Biol Psychiatry, 57*(9), 982-990.

Martin, M. M., & Rubin, R. B. (1995). A new measure of cognitive flexibility. *Psychological Reports, 76*(2), 623-626.

Martin, R. C., & Dahlen, E. R. (2005). Cognitive emotion regulation in the prediction of depression, anxiety, stress, and anger. *Personality and Individual Differences, 39*(7), 1249-1260. doi:10.1016/j.paid.2005.06.004

Martins, B., & Mather, M. (2016). *Default mode network and later-life emotion regulation: Linking functional connectivity patterns and emotional outcomes*.

Martins, B., Sheppes, G., Gross, J. J., & Mather, M. (2016). Age Differences in Emotion Regulation Choice: Older Adults Use Distraction Less Than Younger Adults in High-Intensity Positive Contexts. *Journals of Gerontology*, gbw028.

Mason, M. F., Norton, M. I., Van Horn, J. D., Wegner, D. M., Grafton, S. T., & Macrae, C. N. (2007). Wandering minds: the default network and stimulus-independent thought. *Science, 315*(5810), 393-395.

Maxim, V., Şendur, L., Fadili, J., Suckling, J., Gould, R., Howard, R., & Bullmore, E. (2005). Fractional Gaussian noise, functional MRI and Alzheimer's disease. *Neuroimage, 25*(1), 141-158.

Min, J. A., Yu, J. J., Lee, C. U., & Chae, J. H. (2013). Cognitive emotion regulation strategies contributing to resilience in patients with depression and/or anxiety disorders. *Comprehensive Psychiatry, 54*(8), 1190.

Mitchell, J. P., Banaji, M. R., & Macrae, C. N. (2005). The link between social cognition and self-referential thought in the medial prefrontal cortex. *Journal of cognitive neuroscience, 17*(8), 1306-1315.

Moore, S. A., Zoellner, L. A., & Mollenholt, N. (2008). Are expressive suppression and cognitive reappraisal associated with stress-related symptoms? *Behaviour Research & Therapy, 46*(9), 993-1000.

Morawetz, C., Kellermann, T., Kogler, L., Radke, S., Blechert, J., & Derntl, B. (2016). Intrinsic functional connectivity underlying successful emotion regulation of angry faces. *Soc Cogn Affect Neurosci*, nsw107.

Murphy, E. R., Barch, D. M., Pagliaccio, D., Luby, J. L., & Belden, A. C. (2016). Functional connectivity of the amygdala and subgenual cingulate during cognitive reappraisal of emotions in children with MDD history is associated with rumination. *Dev Cogn Neurosci, 18*, 89-100. doi:10.1016/j.dcn.2015.11.003

Murphy, F. C., Michael, A., & Sahakian, B. J. (2012). Emotion modulates cognitive flexibility in patients with major depression. *Psychological medicine, 42*(7), 1373.

Nashiro, K., Sakaki, M., & Mather, M. (2012). Age Differences in Brain Activity during Emotion Processing: Reflections of Age-Related Decline or Increased Emotion Regulation? Gerontology, 58(2), 156-163.

Nolen-Hoeksema, S. (2000). The role of rumination in depressive disorders and mixed anxiety/depressive symptoms. *Journal of abnormal psychology, 109*(3), 504.

Northoff, G., Heinzel, A., De Greck, M., Bermpohl, F., Dobrowolny, H., & Panksepp, J. (2006). Self-referential processing in our brain—a meta-analysis of imaging studies on the self. *Neuroimage, 31*(1), 440-457.

Ochsner, K. N., Silvers, J. A., & Buhle, J. T. (2012). Functional imaging studies of emotion regulation: a synthetic review and evolving model of the cognitive control of emotion. *Ann N Y Acad Sci, 1251*, E1-24. doi:10.1111/j.1749-6632.2012.06751.x

Park, C., Lazar, N. A., Ahn, J., & Sornborger, A. (2010). A multiscale analysis of the temporal characteristics of resting-state fMRI data. Journal of Neuroscience Methods, 193(2), 334-342.

Paul, S., Simon, D., Kniesche, R., Kathmann, N., & Endrass, T. (2013). Timing effects of antecedent- and response-focused emotion regulation strategies. *Biol Psychol, 94*(1), 136-142. doi:10.1016/j.biopsycho.2013.05.019

Pavlov, S. V., Reva, N. V., Loktev, K. V., Tumyalis, A. V., Korenyok, V. V., & Aftanas, L. I. (2014). The temporal dynamics of cognitive reappraisal: cardiovascular consequences of downregulation of negative emotion and upregulation of positive emotion. *Psychophysiology, 51*(2), 178-186. doi:10.1111/psyp.12159

Phelps, E. A. (2004). Human emotion and memory: interactions of the amygdala and hippocampal complex. *Curr Opin Neurobiol, 14*(2), 198-202.

Power, J. D., Mitra, A., Laumann, T. O., Snyder, A. Z., Schlaggar, B. L., & Petersen, S. E. (2014). Methods to detect, characterize, and remove motion artifact in resting state fMRI. *Neuroimage, 84*, 320-341.

Power, J. D., Schlaggar, B. L., & Petersen, S. E. (2015). Recent progress and outstanding issues in motion correction in resting state fMRI. *Neuroimage, 105*, 536-551.

Quigley, L., & Dobson, K. S. (2014). An examination of trait, spontaneous and instructed emotion regulation in dysphoria. *Cogn Emot, 28*(4), 622-635.

Ragozzino, M. E., Artis, S., Singh, A., Twose, T. M., Beck, J. E., & Jr, M. W. (2012). The selective M1 muscarinic cholinergic agonist CDD-0102A enhances working memory and cognitive flexibility. *Journal of Pharmacology & Experimental Therapeutics, 340*(3), 588-594.

Raz, G., Winetraub, Y., Jacob, Y., Kinreich, S., Maron-Katz, A., Shaham, G., . . . Hendler, T. (2012). Portraying emotions at their unfolding: A multilayered approach for probing dynamics of neural networks. *Neuroimage, 60*(2), 1448-1461.

Ridderinkhof, K. R., Ullsperger, M., Crone, E. A., & Nieuwenhuis, S. (2004). The role of the medial frontal cortex in cognitive control. *Science, 306*(5695), 443-447. doi:10.1126/science.1100301

Roy, A. K., Shehzad, Z., Margulies, D. S., Kelly, A. C., Uddin, L. Q., Gotimer, K., . . . Milham, M. P. (2009). Functional connectivity of the human amygdala using resting state fMRI. *Neuroimage, 2*(45), 614-626.

Sambataro, F., Wolf, N. D., Giusti, P., Vasic, N., & Wolf, R. C. (2013). Default mode network in depression: A pathway to impaired affective cognition. *Clinical Neuropsychiatry, 10*(5), 212-217.

Samson, A. C., Hardan, A. Y., Podell, R. W., Phillips, J. M., & Gross, J. J. (2015). Emotion regulation in children and adolescents with autism spectrum disorder. *Autism Research, 8*(1), 9-18.

Satterthwaite, T. D., Elliott, M. A., Gerraty, R. T., Ruparel, K., Loughead, J., Calkins, M. E., . . . Gur, R. E. (2013). An improved framework for confound regression and filtering for control of motion artifact in the preprocessing of resting-state functional connectivity data. *Neuroimage, 64*, 240-256.

Scheibe, S., Sheppes, G., & Staudinger, U. M. (2015). Distract or reappraise? Age-related differences in emotion-regulation choice. *Emotion, 15*(6), 677.

Shanahan, L., Copeland, W. E., Angold, A., Bondy, C. L., & Costello, E. J. (2014). Sleep problems predict and are predicted by generalized anxiety/depression and oppositional defiant disorder. *Journal of the American Academy of Child & Adolescent Psychiatry, 53*(5), 550-558.

Sheline, Y. I., Barch, D. M., Price, J. L., Rundle, M. M., Vaishnavi, S. N., Snyder, A. Z., . . . Raichle, M. E. (2009). The default mode network and self-referential processes in depression. *Proc Natl Acad Sci U S A, 106*(6), 1942-1947.

Shi, H., Wang, X., Yi, J., Zhu, X., Zhang, X., Yang, J., & Yao, S. (2015). Default mode network alterations during implicit emotional faces processing in first-episode, treatment-naive major depression patients. *Front Psychol, 6*, 1198.

Siegel, J. S., Mitra, A., Laumann, T. O., Seitzman, B. A., Raichle, M., Corbetta, M., & Snyder, A. Z. (2016). Data quality influences observed links between functional connectivity and behavior. *Cerebral Cortex*, 1-11.

Simons, J. S., & Spiers, H. J. (2003). Prefrontal and medial temporal lobe interactions in long-term memory. *Nature Reviews Neuroscience, 4*(8), 637-648.

Spielberger, C. D. (1970). STAI manual for the state-trait anxiety inventory. *Self-Evaluation Questionnaire*, 1-24.

Sripada, C., Angstadt, M., Kessler, D., Phan, K. L., Liberzon, I., Evans, G. W., . . . Swain, J. E. (2014). Volitional regulation of emotions produces distributed alterations in connectivity between visual, attention control, and default networks. *Neuroimage, 89*, 110-121. doi:10.1016/j.neuroimage.2013.11.006

Szymkowicz, S. M., McLaren, M. E., Kirton, J. W., O'shea, A., Woods, A. J., Manini, T. M., . . . Dotson, V. M. (2016). Depressive symptom severity is associated with increased cortical thickness in older adults. *International journal of geriatric psychiatry, 31*(4), 325-333.

Tagliazucchi, E., von Wegner, F., Morzelewski, A., Brodbeck, V., Jahnke, K., & Laufs, H. (2013). Breakdown of long-range temporal dependence in default mode and attention networks during deep sleep. Proc Natl Acad Sci U S A, 110(38), 15419-15424. doi:10.1073/pnas.1312848110

Thiruchselvam, R., Blechert, J., Sheppes, G., Rydstrom, A., & Gross, J. J. (2011). The temporal dynamics of emotion regulation: an EEG study of distraction and reappraisal. *Biol Psychol, 87*(1), 84-92. doi:10.1016/j.biopsycho.2011.02.009

Tozzi, L., Doolin, K., Farrel, C., Joseph, S., O'Keane, V., & Frodl, T. (2017). Functional magnetic resonance imaging correlates of emotion recognition and voluntary attentional regulation in depression: A generalized psycho-physiological interaction study. J Affect Disord, 208, 535-544. doi:10.1016/j.jad.2016.10.029

Uchida, M., Biederman, J., Gabrieli, J. D., Micco, J., de Los Angeles, C., Brown, A., . . . Whitfield-Gabrieli, S. (2015). Emotion regulation ability varies in relation to intrinsic functional brain architecture. *Soc Cogn Affect Neurosci*, nsv059.

Uliaszek, A. A., Zinbarg, R. E., Mineka, S., Craske, M. G., Sutton, J. M., Griffith, J. W., . . . Hammen, C. (2010). The Role of Neuroticism and Extraversion in the Stress-Anxiety and Stress-Depression Relationships. *Anxiety Stress & Coping, 23*(4), 363.

Vanderhasselt, M. A., Baeken, C., Van Schuerbeek, P., Luypaert, R., & De Raedt, R. (2013). Inter-individual differences in the habitual use of cognitive reappraisal and expressive suppression are associated with variations in prefrontal cognitive control for emotional information: an event related fMRI study. *Biol Psychol, 92*(3), 433-439. doi:10.1016/j.biopsycho.2012.03.005

Volokhov, R. N., & Demaree, H. A. (2010). Spontaneous emotion regulation to positive and negative stimuli. *Brain Cogn, 73*(1), 1-6.

Von, W. F., Laufs, H., & Tagliazucchi, E. (2018). Mutual information identifies spurious Hurst phenomena in resting state EEG and fMRI data. *Physical Review E, 97*(2-1), 022415.

Wang, K., Wei, D., Yang, J., Xie, P., Hao, X., & Qiu, J. (2015). Individual differences in rumination in healthy and depressive samples: association with brain structure, functional connectivity and depression. Psychol Med, 45(14), 2999-3008. doi:10.1017/S0033291715000938

Wei, M., Qin, J., Yan, R., Bi, K., Liu, C., Yao, Z., & Lu, Q. (2015). Association of resting-state network dysfunction with their dynamics of inter-network interactions in depression. *J Affect Disord, 174*, 527-534.

Wei, M., Qin, J., Yan, R., Li, H., Yao, Z., & Lu, Q. (2013). Identifying major depressive disorder using Hurst exponent of resting-state brain networks. *Psychiatry Res, 214*(3), 306-312. doi:10.1016/j.pscychresns.2013.09.008

Wingenfeld, K., Wolf, S., Krieg, J. C., & Lautenbacher, S. (2011). Working memory performance and cognitive flexibility after dexamethasone or hydrocortisone administration in healthy volunteers. *Psychopharmacology, 217*(3), 323-329.

Wink, A. M., Bernard, F., Salvador, R., Bullmore, E., & Suckling, J. (2006). Age and cholinergic effects on hemodynamics and functional coherence of human hippocampus. Neurobiol Aging, 27(10), 1395-1404. doi:10.1016/j.neurobiolaging.2005.08.011

Xie, X., Mulej, B. S., Schmid, G., Meng, C., Doll, A., Wohlschläger, A., . . . Pekrun, R. (2016). How do you make me feel better? Social cognitive emotion regulation and the default mode network. *Neuroimage, 134*, 270.

Xu, Y., Zhuo, C., Qin, W., Zhu, J., & Yu, C. (2015). Altered Spontaneous Brain Activity in Schizophrenia: A Meta-Analysis and a Large-Sample Study. *Biomed Research International, 2015*(3), 1-11.

Yan, C.-G., Wang, X.-D., Zuo, X.-N., & Zang, Y.-F. (2016). DPABI: data processing & analysis for (resting-state) brain imaging. *Neuroinformatics, 14*(3), 339-351.

Yan, L., Zhuo, Y., Ye, Y., Xie, S. X., An, J., Aguirre, G. K., & Wang, J. (2010). Physiological origin of low-frequency drift in blood oxygen level dependent (BOLD) functional magnetic resonance imaging (fMRI). Magnetic Resonance in Medicine, 61(4), 819-827.

Yang, A. C., Huang, C. C., Yeh, H. L., Liu, M. E., Hong, C. J., Tu, P. C., . . . Lin, C. P. (2013). Complexity of spontaneous BOLD activity in default mode network is correlated with cognitive function in normal male elderly: a multiscale entropy analysis. Neurobiology of Aging, 34(2), 428-438.

Yang, A. C., & Tsai, S. J. (2013). Is mental illness complex? From behavior to brain. Prog Neuropsychopharmacol Biol Psychiatry, 45, 253-257. doi:10.1016/j.pnpbp.2012.09.015

Zang, Y., Jiang, T., Lu, Y., He, Y., & Tian, L. (2004). Regional homogeneity approach to fMRI data analysis. *Neuroimage, 22*(1), 394-400. doi:10.1016/j.neuroimage.2003.12.030

Zetsche, U., D'Avanzato, C., & Joormann, J. (2012). Depression and rumination: relation to components of inhibition. *Cogn Emot, 26*(4), 758-767. doi:10.1080/02699931.2011.613919

Zou, Q. H., Zhu, C. Z., Yang, Y., Zuo, X. N., Long, X. Y., Cao, Q. J., . . . Zang, Y. F. (2008). An improved approach to detection of amplitude of low-frequency fluctuation (ALFF) for resting-state fMRI: fractional ALFF. *J Neurosci Methods, 172*(1), 137-141. doi:10.1016/j.jneumeth.2008.04.012
