# Supplementary material for: Temporal dynamics of spontaneous default-mode network activity mediate the association between reappraisal and depression
Source: Soc Cogn Affect Neurosci. 2018 Oct 19;13(12):1235–47. doi: 10.1093/scan/nsy092 (PMC6277739; doi:10.1093/scan/nsy092)
Supplement: Supplementary Data [file nsy092_supp.zip › scan-18-121-File011.docx]

**Individual Difference Measures**

Individual differences in everyday use of reappraisal measures were administered before fMRI scanning. We controlled for individual differences in emotion reactivity by assessing neuroticism and trait anxiety and controlled for the use of another common strategy to down regulate emotion by assessing suppression. The primary measure of interest was the reappraisal scale of the ERQ (2). This scale consists of ten items designed to assess individual differences in reappraisal (six) use (e.g., “I control my emotions by changing the way I think about the situation I’m in”). This scale previously has been shown to have good internal consistency and test-retest reliability and to be independent from intelligence and socioeconomic statues (2). Control measures were also administered including: 1) the Chinese-version of 48-item Neuroticism questionnaire of the NEO Five Factor Personality Inventory (6) which assesses an individual’s preference to experience psychological distress; 2) the trait version of the State Trait Anxiety Inventory (STAI trait version) (5) which assesses relatively stable individual differences in anxiety proneness. 3) The suppression scale of the ERQ which assesses individual differences in the use of suppression.

Preprocessing was performed using the Data Processing Assistant for Resting-State fMRI (DPARSFA v3.2, <http://rfmri.org/DPARSF>) and SPM8 (Wellcome Department, University College of London, UK) software based on Matlab (R2010a, processing MathWorks, Inc., USA). DPARSF was used for the following steps: To allow for scanner calibration and participants’ adaptation to the scan, the first 10 volumes were discarded. The remaining volumes were further analyzed. Processing steps included slice timing, head-motion correction, spatial normalization in the Montreal Neurological Institute (MNI) space, and resampling with a 3 × 3 × 3mm^3^ resolution. Participants with head motion > 2.0 mm of translation or > 2.0° of rotation in any direction were excluded from further processing. The linear trend of the fMRI data was removed.

For Hurst exponent, possible variances of DMN_ICA_ and DMN_ROI_ were further removed through linear regressions, which include six head motion parameters and the effect of the signals from a cerebrospinal fluid (CSF) region, a white matter (WM) region and the global brain signal. Further analyses were conducted using Matlab (R2010a, processing MathWorks, Inc., USA), and the Hurst exponent was calculated from DMN_ICA_ and DMN_ROI_ using the WLBMF toolbox (https:// [www.irit.fr/~Herwig.Wendt/](http://www.irit.fr/~Herwig.Wendt/)). The power spectrum of a signal with Hurst exponent H is given by S(f)~f1-2H this could be used to obtain a spectral estimate of H.

A better alternative for the spectral estimation of H is the use of wavelets. We briefly describe the procedure for wavelet H estimation. A wavelet basis is a family of functions φj,k fulfilling the equality, φj,k = 2-j/2φ(2-j-k); This transformation scales the central frequency of φ by a 2-j factor and translates the temporal origin by 2jk. Any signal can be written as a (possibly infinite) linear combination of wavelets and scaling functionsθi,j, with the coefficient associated withφj,k noted as dj,k. An important property characterizing a family of wavelets is the number of vanishing moments N, resulting in the vanishing integrals, ∫tkφ(t)dt = 0; These vanishing integrals render the wavelet decomposition (and therefore the estimation of H) insensitive to polynomial trends of degree smaller than N, a fact following from the orthogonality of the wavelet functionsφj,k and the linear expansion of the signal in terms of these functions. Finally, the variance of the coefficients dj,k of a process with long-range temporal dependence behaves as a power-law, Var(dj,k)~2fe/42j(1-2H); We estimated H voxel-wise using the Wavelet method implemented in the WLBMF toolbox (https:// [www.irit.fr/~Herwig.Wendt/](http://www.irit.fr/~Herwig.Wendt/)). The wavelet basis consisted of Daubechies wavelets with N = 2 vanishing moments. The scales 2 ≤ j ≤ 6 were considered. Therefore, for this choice of wavelets the frequency range under consideration is approximately 0.016 – 0.063 Hz.

For ReHo, the band-pass filtering (0.01 – 0.08 Hz) was conducted to discard highfrequency physiological noise and the frequency drift lower than 0.01 Hz (1). The Resting State fMRI Data Analysis Toolkit (REST, http://rest.restfmri.net) 1.8 (4) was then used for the following steps: Individual ReHo map was generated by calculating the KCC of the time series of a given voxel with those of its neighbors (26 voxels) in a voxel-wise way (8, 9). Afterwards, a wholebrain mask was adopted to remove the non-brain tissues. For standardization purposes, the individual ReHo maps were divided by their own global mean KCC within the whole-brain mask. Then spatial smoothing was performed on the standardized individual ReHo map with a Gaussian kernel of 8 mm full-width at half maximum (FWHM) (7). After the preprocessing, fALFF were computed as previously described (3, 10). First, the resampled images were smoothed with a Gaussian kernel of 8 mm. Then the frequency band filtering was set as 0.01 – 0.08 Hz, and the time courses were converted to the frequency band using a Fast Fourier Transform.

**Table 1 fALFF/ReHo of DMN and individual differences in reappraisal/suppression**

|  | Reappraisal | Suppression |
| --- | --- | --- |
| fALFF of MPFC | r = -0.006; p = 0.953 | r = 0.153.; p=0.119 |
| fALFF of PCC | r = 0.095; p = 0.337 | r = 0.074; p = 0.451 |
| fALFF of MTL | r = 0.096; p = 0.328 | r = 0.065; p = 0.507 |
| ReHo of MPFC | r = -0.012; p = 0.906 | r = 0.025; p = 0.798 |
| ReHo of PCC | r = -0.018; p = 0.856 | r = 0.106; p = 0.283 |
| ReHo of MTL | r = 0.026; p = 0.793 | r = -0.084; p = 0.397 |

Spearman rank-correlations and scatter-plots displaying the relationship between the scores of habitual use of reappraisal/suppression and fALFF/ReHo of DMN

We use Structural Equation Modeling (SEM) to test the effect of gender difference on the mediation model, multiple groups comparison result showed that there is no significant effect (df=3, △X^2^=0.209, p=0.976 ). Then we use partial correlation analysis to exam effects of sex on reappraisal, depressive and and Hurst exponent. We add this part in the supplementary materials. The results are shown below:

**Table2 Partial correlations between all variables controlling for gender (N = 105)**

|  | Reappraisal | Depression |
| --- | --- | --- |
| H of DMN_ICA_ | r = -0.319; p = 0.001 | r = 0.301; p = 0.001 |
| H of DMN_ROI_ | r = -0.307; p = 0.002 | r = 0.308; p = 0.002 |

**Table 3** **Mediation effects of H of DMN in the prediction of Depression controlling for gender (N= 105).**

| Mediator | Depression | |
| --- | --- | --- |
|  | Point Estimate (a*b) | Bootstrapping BC 95% CI |
| H of DMN_ICA_ | -0.1598 | [-0.3662, -0.0226] |
| Gender _ICA_ | -0.9447 | [-3.9884, 2.0989] |
| H of DMN_ROI_ | -0.1499 | [-0.3767, -0.0257] |
| Gender _ROI_ | -0.7427 | [-3.7774, 2.2919] |

**Note:** The mediation effects of reappraisal on subjective depression controlling for gender were bootstrapped using 5000 samples. Ninety-five percent bias-corrected confidence intervals for all indirect effects and contrasts were generated.

**Mediation analysis**

| Mediator | Depression | |
| --- | --- | --- |
|  | Point Estimate (a*b) | Bootstrapping BC 95% CI |
| H of DMN_ICA_ | -0.0718 | [-0.1651, -0.0112] |
| H of DMN_ROI_ | -0.0691 | [-0.1706, -0.0105] |
| fALFF of DMN | 0.0020 | [-0.0179, 0.0509] |
| ReHo of DMN | 0.0101 | [-0.0128, 0.0790] |
| H of PCC | -0.0180 | [-0.0818, 0.0306] |
| fALFF of PCC | -0.0048 | [-0.0687, 0.0156] |
| ReHo of PCC | 0.0023 | [-0.0316, 0.0474] |
| H of MPFC | -0.0788 | [-0.1897, -0.0175] |
| fALFF of MPFC | 0.0015 | [-0.0168, 0.0489] |
| ReHo of MPFC | 0.0026 | [-0.0260, 0.0500] |
| H of MTL | 0.0218 | [-0.0147, 0.0871] |
| fALFF of MTL | -0.0052 | [-0.0736, 0.0117] |
| ReHo of MTL | -0.0070 | [-0.0614, 0.0129] |

Note: The mediation effects of the use of reappraisal on subjective depression were bootstrapped using 5000 samples. Ninety-five percent bias-corrected confidence intervals for all indirect effects and contrasts were generated.

1. Greicius MD, Krasnow B, Reiss AL, Menon V. Functional connectivity in the resting brain: A network analysis of the default mode hypothesis. Proc Natl Acad Sci U S A. 2003;100(1):253-8.

2. Gross JJ, John OP. Individual differences in two emotion regulation processes: Implications for affect, relationships, and well-being. Journal of Personality and Social Psychology. 2003;85(2):348-62.

3. Haag LM, Heba S, Lenz M, Glaubitz B, Hoffken O, Kalisch T, et al. Resting BOLD fluctuations in the primary somatosensory cortex correlate with tactile acuity. Cortex. 2015;64:20-8.

4. Song XW, Dong ZY, Long XY, Li SF, Zuo XN, Zhu CZ, et al. REST: a toolkit for resting-state functional magnetic resonance imaging data processing. Plos One. 2011;6(9):e25031.

5. Spielberger CD. STAI manual for the state-trait anxiety inventory. Self-Evaluation Questionnaire. 1970:1-24.

6. Szymkowicz SM, McLaren ME, Kirton JW, O'shea A, Woods AJ, Manini TM, et al. Depressive symptom severity is associated with increased cortical thickness in older adults. International journal of geriatric psychiatry. 2016;31(4):325-33.

7. Wang L, Li K, Zhang Q, Zeng Y, Dai W, Su Y, et al. Short-term effects of escitalopram on regional brain function in first-episode drug-naive patients with major depressive disorder assessed by resting-state functional magnetic resonance imaging. Psychological Medicine. 2014;44(7):1417.

8. Z L, A K, J P, J D, Z W. Test-retest stability analysis of resting brain activity revealed by blood oxygen level-dependent functional MRI. Journal of Magnetic Resonance Imaging. 2012;36(2):344.

9. Zang Y, Jiang T, Lu Y, He Y, Tian L. Regional homogeneity approach to fMRI data analysis. Neuroimage. 2004;22(1):394-400.

10. Zou QH, Zhu CZ, Yang Y, Zuo XN, Long XY, Cao QJ, et al. An improved approach to detection of amplitude of low-frequency fluctuation (ALFF) for resting-state fMRI: fractional ALFF. J Neurosci Methods. 2008;172(1):137-41.
